# Genome-Wide Identification and Expression Analyses of the *FAR1/FHY3* Gene Family Provide Insight into Inflorescence Development in Maize

**DOI:** 10.3390/cimb46010027

**Published:** 2024-01-02

**Authors:** Huaijun Tang, De Jing, Cheng Liu, Xiaoqing Xie, Lei Zhang, Xunji Chen, Changyu Li

**Affiliations:** 1 Institute of Grain Crops, Xinjiang Academy of Agricultural Sciences, Urumqi 830091, China; tanghuaijun83@sina.com (H.T.); liuchengxj@126.com (C.L.); 13565990757@163.com (X.X.); 18999224030@163.com (L.Z.); 2 Key Laboratory of Genome Research and Genetic Improvement of Xinjiang Characteristic Fruits and Vegetables, Institute of Horticultural Crops, Xinjiang Academy of Agricultural Sciences, Urumqi 830091, China; 17608476967@163.com; 3 Institute of Nuclear and Biotechnology, Xinjiang Academy of Agricultural Sciences, Urumqi 830091, China

**Keywords:** *ZmFAR1*, transcription factors, far-red light, causal variation, maize

## Abstract

As transcription factors derived from transposase, *FAR-RED IMPAIRED RESPONSE1* (*FAR1*) and its homolog *FHY3* play crucial roles in the regulation of light signaling and various stress responses by coordinating the expression of downstream target genes. Despite the extensive investigation of the FAR1/FHY3 family in *Arabidopsis thaliana* and other species, a comprehensive examination of these genes in maize has not been conducted thus far. In this study, we employed a genomic mining approach to identify 16 *ZmFAR1* genes in the maize inbred line B73, which were further classified into five subgroups based on their phylogenetic relationships. The present study characterized the predicted polypeptide sequences, molecular weights, isoelectric points, chromosomal distribution, gene structure, conserved motifs, subcellular localizations, phylogenetic relationships, and cis-regulatory elements of all members belonging to the ZmFAR1 family. Furthermore, the tissue-specific expression of the 16 *ZmFAR1* genes was analyzed using RNA-seq, and their expression patterns under far-red light conditions were validated in the ear and tassel through qRT-qPCR. The observed highly temporal and spatial expression patterns of these *ZmFAR1* genes were likely associated with their specific functional capabilities under different light conditions. Further analysis revealed that six *ZmFAR1* genes (*ZmFAR1-1*, *ZmFAR1-10*, *ZmFAR1-11*, *ZmFAR1-12*, *ZmFAR1-14*, and *ZmFAR1-15*) exhibited a response to simulated shading treatment and actively contributed to the development of maize ears. Through the integration of expression quantitative trait loci (eQTL) analyses and population genetics, we identified the presence of potential causal variations in *ZmFAR1-14* and *ZmFAR1-9*, which play a crucial role in regulating the kernel row number and kernel volume weight, respectively. In summary, this study represents the initial identification and characterization of ZmFAR1 family members in maize, uncovering the functional variation in candidate regulatory genes associated with the improvement of significant agronomic traits during modern maize breeding.

## 1. Introduction

Light is crucial for plant growth [1,2], significantly affecting development under canopy shade. Plants use sophisticated photoreceptors to detect light changes [3] and adapt their growth strategies. The FAR1/FHY3 gene family plays a key role in plant light signaling and shade adaptation [4], with far-red light activating the *FAR1/FHY3* genes to enhance the phyA-mediated high light responses [5,6], aiding in light detection and growth. The FAR1/FHY3 proteins contain three domains: the C2H2 zinc finger, core transposase, and SWIM zinc finger domains [7,8]. Arabidopsis has 14 identified *FAR1/FHY3* genes, phylogenetically classified into five groups, encoding sequences of 531 to 851 amino acids, with specific functions across tissues and developmental stages [5,7].

*FAR1* and *FHY3* are multifunctional genes studied in plants, known to upregulate ELF4 and influence flowering by connecting light signals to the miR156-SPL aging pathway, as well as to regulate starch synthesis by activating ISA2 [9,10,11,12]. In *far1* and *fhy3* mutants, the lack of *CCA1* expression suggests that these genes also affect light-induced processes [10]. Additionally, *FAR1/FHY3* interact with jasmonic acid pathways to balance growth and defense [12,13] and affect branching through *SPL9/SPL15* interactions and strigolactone signaling [14]. These genes also play roles in chlorophyll production, chloroplast division, ABA signaling, and delaying leaf senescence by integrating light and ethylene signals [15,16]. FHY3’s extensive targets imply its broad impact on plant development [6].

In addition, the upregulation of *THERMO-SENSITIVE BARREN PANICLE* (*TAP*), a member of the FAR1 family in rice, might be induced by elevated temperatures. This up-regulation is believed to modulate the proper growth and development of rice inflorescences and spikelets through the formation of protein complexes with *OsYABBY4/5* [17]. Additionally, several *AhFAR1* genes have been detected in peanuts, suggesting their potential involvement in regulating pod development [18]. Moreover, the dispersed expansion of FAR1/FRS-like genes has been observed in *Rosa wichuraiana* ‘Basye’s Thornless’, and their diverse expression patterns have been linked to the control of shoot growth and the flowering time [19]. Despite the numerous attributions assigned to these genes, there remains a dearth of systematic inquiries into this gene family in maize. Consequently, it is imperative to ascertain the constituents of the ZmFAR1 family in maize and comprehend their responses to shading (light conditions enriched in far-red light), along with their prospective functions in governing plant structural development.

Maize is a major global crop facing challenges such as reduced productivity due to shade avoidance syndrome (SAS) under high-density planting, which is further influenced by decreased red/far-red light [20,21]. In this study, a comprehensive analysis was conducted to identify ZmFAR1 family members in the maize B73 genome. The investigation included an examination of the physicochemical properties, sequence characteristics, conserved domains, and phylogenetic relationships of 16 *ZmFAR1* genes. Furthermore, the subcellular localization of maize genes was predicted, and analyses of the cis-regulatory elements and miRNA–mRNA regulatory networks were performed to gain insights into potential gene functions. The expression patterns of the *ZmFAR1* genes were analyzed using RNA-seq data and the expression levels were further validated through qRT-PCR experiments in the ear and tassel at various developmental stages under simulated shading conditions. In addition, we endeavored to ascertain the potential causal variations and favorable haplotypes for ZmFAR1 family members through the integration of eQTL analyses and population genetics. Our discoveries might establish a theoretical framework for future investigations into the biological functions of ZmFAR1 transcription factor family members and provide valuable perspectives on the genetic enhancement of maize inbred lines during modern maize breeding.

## 2. Materials and Methods

### 2.1. Identification of ZmFAR1 Genes in Maize B73

The FAR1 gene family’s Hidden Markov Models (HMMs) PF03101, PF10551, and PF04434 were obtained from the Pfam protein family database (https://pfam.xfam.org/) (accessed on 2 April 2023). The maize B73 genome sequence, gff3 annotation, and protein sequence files were obtained from the Maize Genome Database (https://www.maizegdb.org/) (accessed on 2 April 2023). The protein sequences were scanned using hmmsearch in the HMMER (v3.3.2) software, and those with an E-value < 1 × 10^−5^ were treated as criteria for the screening of high-quality FAR1 proteins [22]. The FAR1 protein sequences of *Arabidopsis thaliana* were also retrieved from the TAIR database (https://www.arabidopsis.org/) (accessed on 3 April 2023). The BLASTP (v2.9.0) comparison tool was utilized for the identification of FAR1 homologous genes in maize [23]. The BLASTP results were integrated with the hmmsearch results, and the intersections were taken as candidate ZmFAR1 family members. Finally, protein domain prediction was performed via the Interpro (https://www.ebi.ac.uk/interpro, accessed on 7 February 2023) and SMART online tools (http://smart.embl-heidelberg.de/) to perform further verification (accessed on 5 April 2023). The physicochemical properties and subcellular localizations of *ZmFAR1* genes were analyzed through ExPASy (http://www.expasy.org) and WoLFPSORT (https://wolfpsort.hgc.jp/), respectively (accessed on 7 April 2023). The secondary structure was also predicted through SOPMA (https://npsa-prabi.ibcp.fr/cgi-bin/npsa_automat.plpage=npsa_sopma.html) (accessed on 8 April 2023).

### 2.2. Phylogenetic Analyses of ZmFAR1 Genes

Multiple sequence alignment of the FAR1 protein sequences of maize B73, *Sorghum bicolor*, and *Arabidopsis thaliana* was performed using the muscle (v3.8.31) program with default parameters [24]. Based on the alignment results, the optimal model was validated, and the maximum-likelihood approach was used to create the phylogenetic tree using iqtree (v1.6.12); the bootstrap value was set to 1000 [25]. Using the web application iTOL, the phylogenetic tree was visualized and rendered (https://itol.embl.de/) (accessed on 10 April 2023).

### 2.3. Gene Structure and Conserved Motif Analysis of ZmFAR1 Genes

The *ZmFAR1* gene structures were investigated through GSDS (http://gsds.cbi.pku.edu.cn/) (accessed on 11 April 2023) [26]. Using web-based MEME, the conserved motifs in ZmFAR1 family proteins were predicted. The number of motifs was set to 15 (https://meme-suite.org/meme) (accessed on 12 April 2023) [27]. The evolutionary tree, exon–intron structures, and conserved motifs were all visualized by the TBtools program [28].

### 2.4. Chromosomal Location and Collinearity Analysis of ZmFAR1 Genes

Based on the genetic information and length information of maize chromosomes, all distributions of *ZmFAR1* genes were mapped using MapChart (v2.3.2). The collinearity of FAR1 family members among *Sorghum bicolor*, maize B73, and *Oryza sativa* was analyzed using MCScanX with default parameters and visualized by TBtools [28,29].

### 2.5. Cis-Acting Elements and Interaction Network Analysis of ZmFAR1 Genes

The upstream sequences (2000 bp) of all *ZmFAR1* genes were extracted to perform cis-acting element analysis by uploading to the PlantCARE database (http://bioinformatics.psb.ugent.be/webtools/plantcare/html/) (accessed on 13 April 2023). Using the R program ggplot2, the distributions and functions of all anticipated cis-regulatory elements in the promoters of all genes were studied and visualized [30]. The psRNAtarget online platform was used to predict the microRNAs (miRNAs) that target *ZmFAR1*. The expectation was set to 5 and no gaps were permitted (https://www.zhaolab.org/psRNATarget/) (accessed on 15 April 2023). Cytoscape was used to provide a visual representation of the interaction network.

### 2.6. Tissue-Specific Expression Analysis of ZmFAR1 Genes

Using the NCBI SRA database, we obtained transcriptome data for the ear, leaf tip, tassel, leaf base, leaf center, embryo, root, shoot, anther, and endosperm of maize B73 (http://www.ncbi.nlm.nih.gov/sra/) with the accession number ERR3773807-ERR3773827 (accessed on 20 April 2023). All raw sequencing data were first preprocessed using fastp (v0.20.1) [31], followed by mapping to the reference genome using HISAT2 [32]. Cufflinks and Cuffcompare were utilized to assemble and annotate the predicted transcripts, respectively [33]. After normalizing the gene counts, the expression levels of all expressed genes were obtained, from which the expression levels of *ZmFAR1* genes were extracted and visualized using the R package ggplot2 [30]. The transcriptome data for the far-red and shade treatment of maize B73 were obtained from the Maize RNA-seq Database (http://ipf.sustech.edu.cn/pub/zmrna/download/, accessed on 20 January 2023).

### 2.7. Plant Materials and Stress Treatments

The seedlings of the maize inbred line B73 were grown in an incubator at an ambient temperature of 27 °C. The intensity of white light was set to 330 μmol m^−2^ s^−1^ and the R:FR ratio was 14.3. The photoperiod was adjusted to 12 h light/12 h dark. The control group was defined as follows: the seedlings of maize B73 were grown under white light until the sampling period. The following criteria were used to define the treatment groups: the seedlings of maize B73 were grown in a simulated shade treatment after the seedlings were grown until stage V2 and then treated with 21 μmol m^−2^ s^−1^ far-red light for 30 min at the end of each day (EOD-FR treatment). The ear and tassel were collected at stages V8 to V11 and V8 to V12, respectively. After the samples were collected, they were promptly frozen in liquid nitrogen and stored in a −80 °C freezer until they were utilized for RNA extraction.

### 2.8. RNA Isolation and Quantitative qRT-PCR Analysis

Total RNA was extracted from samples using an RNAsimple Total RNA Kit (Tiangen, Beijing, China). First, cDNAs were reverse-transcribed from the total mRNAs using the Hifair^®^ III 1st Strand cDNA Synthesis SuperMix (Yeasen Biotechnology, Shanghai, China). Then, quantitative real-time PCR (qRT-PCR) was performed using Hieff UNICON^®^ qPCR SYBR Green Master Mix (Yeasen Biotechnology, Shanghai, China) on an Applied Biosystems 7500 real-time PCR system. The expression levels of *ZmFAR1* genes were calculated by the 2^−ΔCT^ method and normalized against the reference gene ACTIN [34]. Values were calculated as the means ± standard deviations (SD) of three biological replicates. All primer sequences are listed in Supplementary Table S4.

### 2.9. Expression Quantitative Trait Loci (eQTL) Analysis

We performed eQTL analysis based on high-quality SNPs and gene expression levels in the ear in a previously reported study and using the LMM model in the EMMAX software emmax-intel64-20120205 [35,36]. The method of cis-eQTL identification was similar to a previous study, with minor modifications [35].

### 2.10. Statistical Analysis

Data processing with simple calculations (mean and standard deviation) and Microsoft Excel (version 16.01) were used to perform Student’s *t*-tests. The *p*-value < 0.05 was considered statistically significant between the control and treatment (* *p*-value < 0.05, ** *p*-value < 0.01).

## 3. Results

### 3.1. Identification and Chromosomal Location of ZmFAR1 Genes in Maize

**Figure 1:**
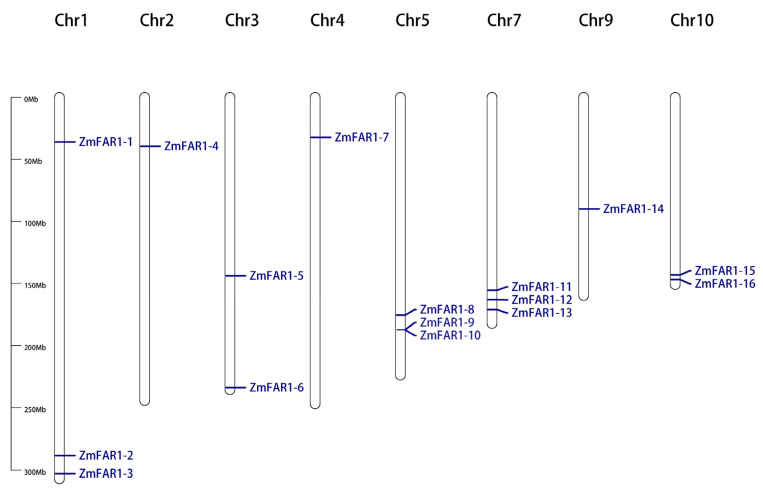
Distribution of ZmFAR1 family members in maize B73.

In the maize inbred line B73 genome, 16 putative *ZmFAR1* genes were found and named *ZmFAR1-1* to *ZmFAR1-16* based on their chromosomal locations. Based on the genomic annotation information of maize inbred line B73, all 16 *ZmFAR1* genes were unevenly distributed throughout the eight chromosomes (Figure 1). Specifically, *ZmFAR1-1*, *ZmFAR1-2*, and *ZmFAR1-3* were located on chromosome 1 (Chr1); *ZmFAR1-8*, *ZmFAR1-9*, and *ZmFAR1-10* were located on Chr5; and *ZmFAR1-11*, *ZmFAR1-12*, and *ZmFAR1-13* were located on Chr7. Chr3 and Chr10 each contained two gene members (*ZmFAR1-5* and *ZmFAR1-6*; *ZmFAR1-15* and *ZmFAR1-16*), and Chr2, Chr4, and Chr9 contained *ZmFAR1-4*, *ZmFAR1-7*, and *ZmFAR1-14*, respectively.

### 3.2. Phylogenetic Analysis of ZmFAR1 Genes

**Figure 2:**
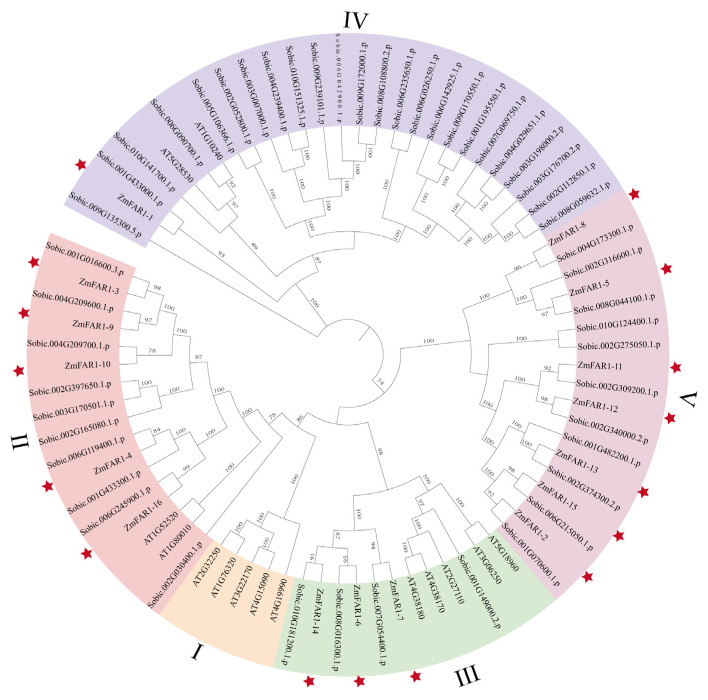
Phylogenetic analysis of maize B73, *Sorghum bicolor*, and *A. thaliana*. The genes labeled with “AT” represent the genes of *Arabidopsis*. The genes labeled with “Zm” represent the genes of maize inbred line B73. The genes labeled with “Sobic” represent the genes of *Sorghum bicolor*. Each position of the *ZmFAR1* gene is noted with an asterisk. All *FAR1* genes were subdivided into groups I through V.

To evaluate the evolutionary relationships among the FAR1 proteins, an unrooted phylogenetic tree of 79 FAR1 protein sequences was constructed using the ML method (Supplementary Table S1). These 79 FAR1 proteins were derived from three species, 14 from *Arabidopsis thaliana* (*A. thaliana*), 49 from *Sorghum bicolor* (*S. bicolor*), and 16 from maize B73. All 79 *FAR1* genes were divided into five subgroups according to the classification of *FAR1* genes in *Arabidopsis*, designated I to V (Figure 2). There were 5 *AtFAR1* members in the I subgroup; 5 *ZmFAR1* members, 2 *AtFAR1* members, and 10 *SbFAR1* members in the II subgroup; 3 *ZmFAR1* members, 5 *AtFAR1* members, and 4 *SbFAR1* members in the III subgroup; 1 *ZmFAR1* member, 2 *AtFAR1* members, and 24 *SbFAR1* members in the IV subgroup; and 7 *ZmFAR1* members and 11 *SbFAR1* members in the V subgroup. However, subgroup I had no *ZmFAR1* members, and subgroup V contained the largest number of ZmFAR1 proteins.

### 3.3. Conserved Structure and Motif Analyses of ZmFAR1 Genes

**Figure 3:**
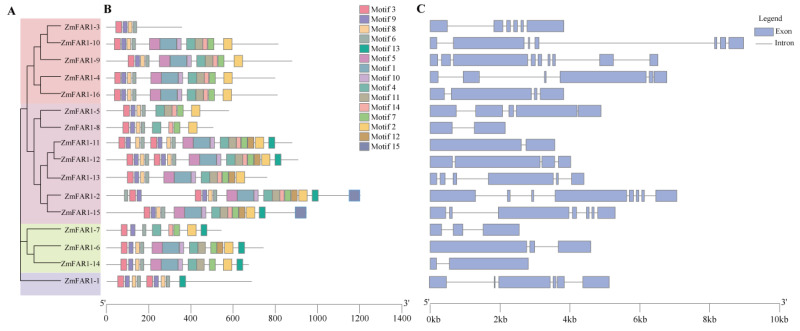
The gene structure and motifs of ZmFAR1 family members in maize B73. (**A**) Phylogenetic tree of *ZmFAR1* genes. (**B**) Conserved motifs of *ZmFAR1* proteins. The order of the motifs corresponds to their position within individual protein sequences. (**C**) The exon–intron organization of *ZmFAR1* genes. Blue boxes represent exons; black lines represent introns. The lengths of exons and introns for each *ZmFAR1* gene are proportionally displayed.

To explore the distribution and structural diversification of the conserved motifs of the ZmFAR1 proteins, we analyzed their conserved motifs and intron–exon structure. The results revealed that all ZmFAR1 proteins in maize B73 featured an N-terminal C2H2 zinc finger domain at the N-terminus with the capability of binding DNA (Supplementary Figure S1). As shown in Figure 3A, the *ZmFAR1* genes with identical conserved motifs were clustered along the same evolutionary branch, suggesting that they might have functional similarities. Then, we further analyzed the conserved DNA motifs, and 15 conserved motifs were identified in the 16 ZmFAR1 proteins (Figure 3B and Figure S2). This result illustrated that the highly conserved *ZmFAR1* genes might share the same structural characteristics. The structure motif3-motif9-motif8-motif6 (FAR1 DNA-binding domain) was found to be present in all family members. Interestingly, *ZmFAR1-1*, *ZmFAR1-11*, and *ZmFAR1-12* contained two C2H2 zinc finger domains, suggesting that they might have unique functions compared to other *ZmFAR1* genes. *ZmFAR1-2* and *ZmFAR1-15* contained all motifs, while *ZmFAR1-3* only contained four motifs. All subgroups except *ZmFAR1-1* and *ZmFAR1-3* contained motif 2, motif 4, and motif 7, while motif 10, motif 12, and motif 13 were only present in subgroups II and IV. In addition, motif 15 appeared only in the IV subgroup. Thus, *ZmFAR1* genes with comparable conserved motifs were clustered in the same evolutionary branch, and this finding was compatible with the phylogenetic relationships (Figure 3A,B).

The exon–intron structures could reveal the lineage-specific features of eukaryotic genes, as they differ significantly among eukaryotic genomes [37]. Therefore, exon–intron organization analysis of the *ZmFAR1* genes was performed to understand the structural diversity and characteristics (Figure 3C). According to the data, *ZmFAR1-9* included the highest number of exons (9), followed by *ZmFAR1-2* (8), *ZmFAR1-10* (7), and *ZmFAR1-15* (7). In contrast, *ZmFAR1-8*, *ZmFAR1-11*, and *ZmFAR1-14* had only two exons and one intron, whereas *ZmFAR1-5* had five exons and four introns. *ZmFAR1-12* and *ZmFAR1-16* each had four exons and three introns. *ZmFAR1-6* and *ZmFAR1-7* both had three exons and two introns. The remaining genes all contained six exons and five introns.

### 3.4. Collinearity Analysis of ZmFAR1 Genes

**Figure 4:**
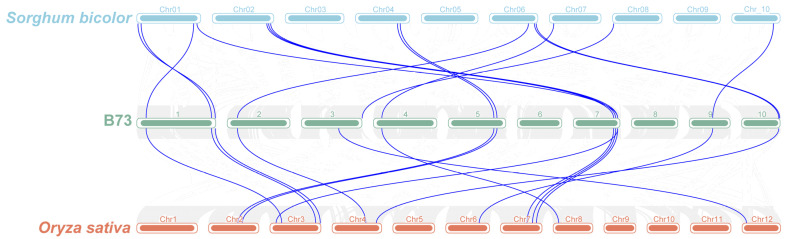
Synteny analysis of FAR1 genes between *Sorghum bicolor*, maize inbred line B73, and Oryza sativa. Gray lines represent all collinearity blocks between genomes, and the colinear *FAR1* gene pairs are highlighted by blue lines. The numbers in the figure represent chromosomes.

Collinearity analysis could provide insights into the divergence and evolutionary history of the genome [38]. To further investigate the covariance of the *FAR1* gene family, we identified collinear relationships among *FAR1* in *S. bicolor*, maize B73, and *A. thaliana* (Figure 4). A total of 15 homologous gene pairs between maize B73 and *S. bicolor* were found, with *ZmFAR1-13* being homologous to two *S. bicolor* genes (*Sobic.0G482200.1* and *Sobic.0G374300.2*). The homologous genes of *ZmFAR1-4*, *ZmFAR1-8*, and *ZmFAR1-9* (*Sobic.0G119400.1*, *Sobic.0G173300.1*, and *Sobic.0G209600.1*) could only be found in *S. bicolor*. There was a total of 14 homologous gene pairs found between maize B73 and *O. sativa*. *ZmFAR1-6* and *ZmFAR1-16* were found to be homologous to *Sobic.0G016300.1* and *Sobic.006G245900.1*, respectively, but no homologous genes were found in *O. sativa*. Only *ZmFAR1-10* had no homologous genes in *S. bicolor* and *O. sativa*. Therefore, studying the evolutionary relationships of the FAR1 family members will aid in the study of *ZmFAR1* gene function.

### 3.5. Cis-Acting Element Analysis of ZmFAR1 Genes

**Figure 5:**
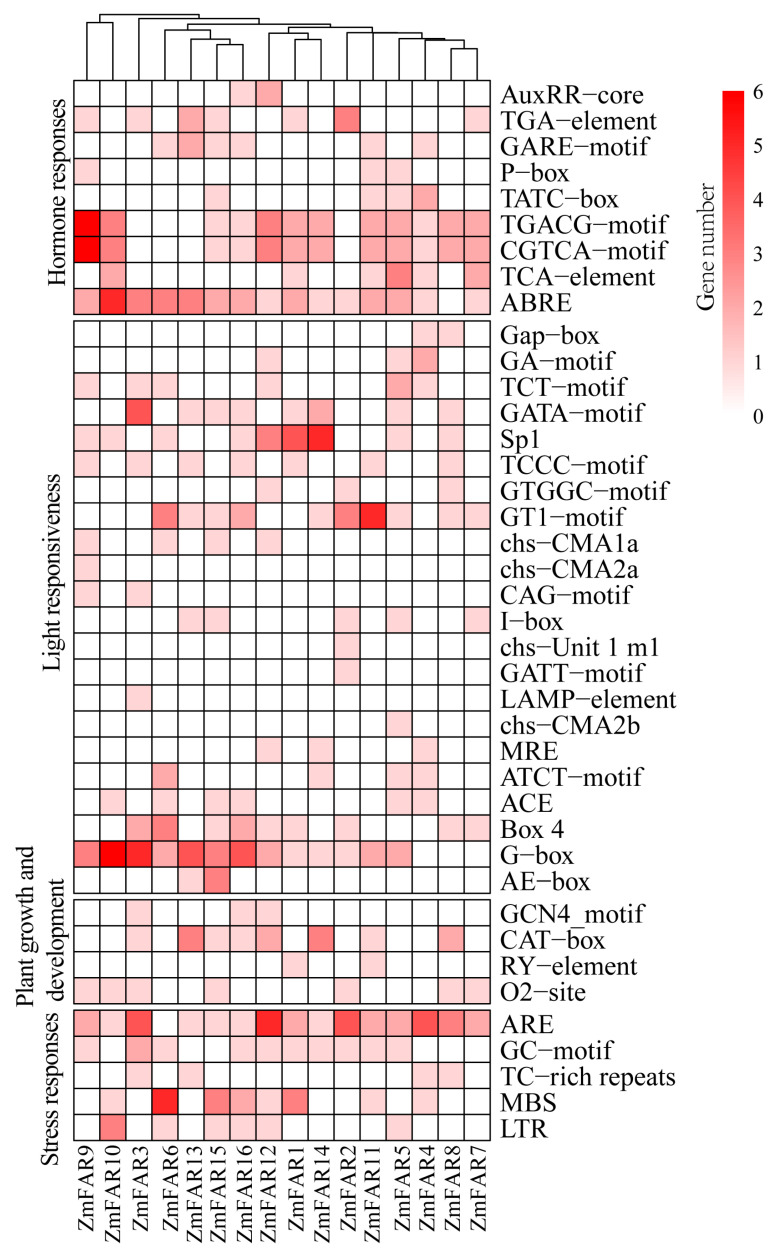
Cis-acting elements in the promoters of ZmFAR1 family members.

Cis-regulatory elements are specific DNA sequences that are located upstream of gene coding sequences, which are specific binding sites for the proteins involved in the initiation and regulation of transcription [39]. To investigate the probable roles and regulation of ZmFAR1 family members, we performed cis-acting element analysis based on the putative promoter sequences in the upstream 2000 bp region of each gene. In total, 374 cis-elements were identified across all *ZmFAR1* genes, which could be categorized into four primary groups, including hormone and stress responses, light responses, and plant growth and development (Figure 5). The majority of the 16 *ZmFAR1* genes included light-responsive cis-elements (40.11%, 150/374), followed by hormone-responsive elements (32.89%, 123/374), stress response elements (20.05%, 75/374), and the lowest proportion of cis-elements (6.95%, 26/374) associated with plant growth and development (Supplementary Table S2). The Gap-box, GA-motif, TCT-motif, GATA-motif, Sp1, TCCC-motif, GTGGC-motif, GT1-motif, chs-CMA1a, CAG-motif, I-box, MRE, ATCT-motif, ACE, Box 4, G-box, and AE-box were all identified as light-responsive cis-elements. Of these, the G-box was the most abundant (24%), followed by the GT1-motif (12.67%) and Sp1 (12%). Hormone-responsive cis-elements included growth hormone-responsive elements (AuxRR-core and TGA-element), gibberellin-responsive elements (GARE motif, TATC-box, and P-box), MeJA-responsive elements (TGACG-motif and CGTCA-motif), and salicylic acid responsiveness (TCA-element and ABRE). Among them, ABRE was the most abundant (25.2%, 31/123), and AuxRR-core and P-box were the least abundant (2.44%, 3/123) among the hormone-responsive cis-elements. The stress-responsive cis-elements comprised 46.67% ARE (anaerobic induction), 22.6% MBS (drought induction), 14.6% GC-motif (hypoxia-specific induction), 10.67% LTR (low-temperature response), and 5.3% TC-rich repeat sequences (defense and stress responsiveness). The plant growth and development cis-elements comprised 11.54% GCN4_motif (cis-regulation of endosperm expression), 53.85% CAT-box (meristem expression), 7.69% RY-elements (seed-specific regulation), and 26.92% O2-site maize alcoholic proteins (metabolic regulation). The investigated cis-elements had the potential to play vital roles in the process of regulating gene expression in response to a variety of stimuli, as well as in the process of plant growth and development. We also found differences in the number of cis-acting elements of each ZmFAR1 family member. *ZmFAR1-12* had the highest number of cis-elements (31 members), followed by *ZmFAR1-3* and *ZmFAR1-9* (28 members), while *ZmFAR1-7* had the lowest number of cis-elements (14 members). These results indicate that the functional differences between different subfamily members may be due to the presence of different numbers of cis-regulatory elements.

### 3.6. Target miRNA Prediction and Interactive Network Analysis of ZmFAR1 Genes

**Figure 6:**
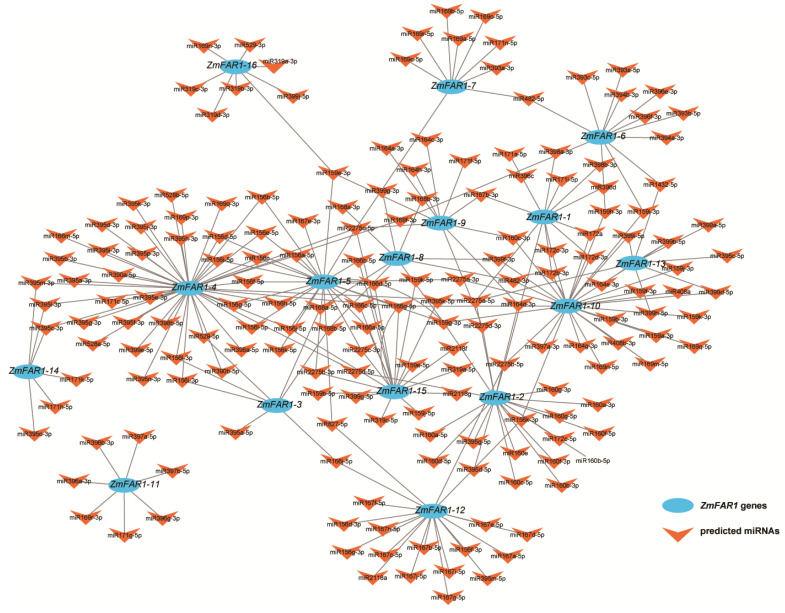
Predicted miRNA–mRNA regulatory network. Gray lines represent the interactions between miRNAs and *ZmFAR1* genes. The blue circles represent *ZmFAR1* genes, and the red arrowheads represent miRNAs.

miRNAs control multiple cellular and biological processes by regulating gene expression. To predict the downstream targets of the *ZmFAR1* genes, we constructed a miRNA–mRNA coexpression network. The results showed that there was a potential targeting relationship between 179 miRNAs and 16 *ZmFAR1* genes (Supplementary Table S3). *ZmFAR1-4*, *ZmFAR1-5*, and *ZmFAR1-10* were the primary targets of 84 miRNAs from 20 miRNA families. In particular, *ZmFAR1-4* was targeted by the highest number of miRNAs (45 members) from 12 miRNA families (Figure 6). *ZmFAR1-10*, *ZmFAR1-5*, *ZmFAR1-2*, *ZmFAR1-15*, and *ZmFAR1-12* were targeted by 31, 26, 20, 20, and 19 miRNAs, respectively. From the miRNA viewpoint, the miR2275 family could target 10 *ZmFAR1* genes, followed by miR159 (eight target *ZmFAR1* genes) and miR395 (seven target *ZmFAR1* genes). Previous studies showed that miR156 could regulate flowering, and we found that the miR156 family targets six *ZmFAR1* genes, which may also regulate plant flowering. Furthermore, six miRNAs (miR396a-3p, miR396b-3p, miR396g-3p, miR169r-3p, miR171g-5p, miR397a-5p, and miR397b-5p) could only target *ZmFAR1-11*, suggesting that this gene might play a different role compared to the other *ZmFAR1* family members.

### 3.7. Expression Analysis of ZmFAR1 Genes

**Figure 7:**
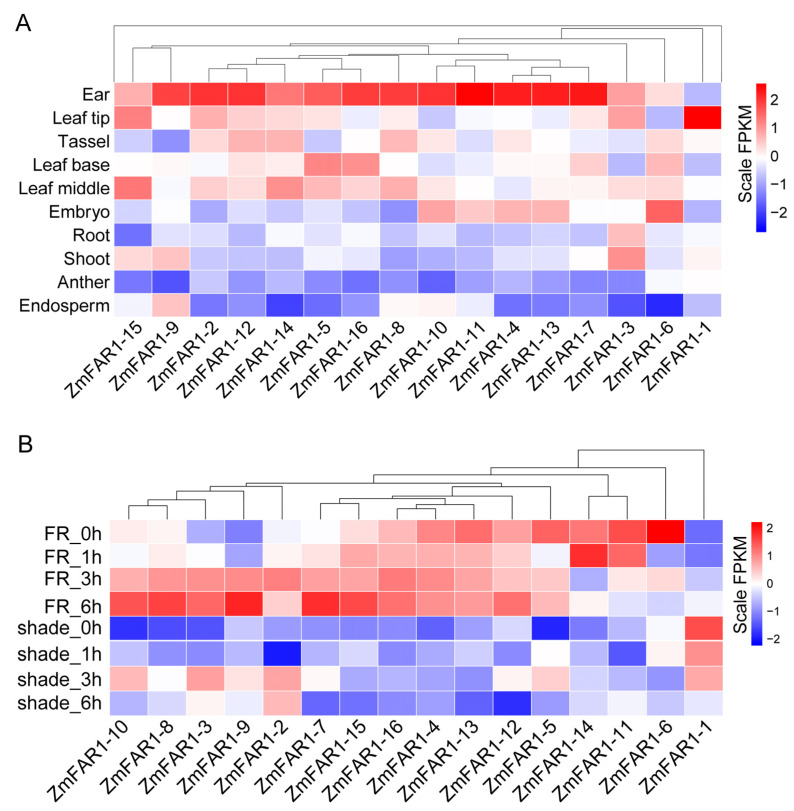
The expression profiles of ZmFAR1 family members in different tissues (**A**) and under different light treatment conditions (**B**). FR refers to far-red light treatment under 6 µmol/m^2^s far-red light. Shade refers to the simulated shade treatment under blue, 15 µmol/m^2^s; red, 12 µmol/m^2^s; far-red, 105 µmol/m^2^s.

Gene expression patterns provide important clues in understanding potential gene functions. To evaluate the transcriptome properties and biological activities of all *ZmFAR1* genes, we analyzed the expression patterns of these genes using public transcriptome data in different B73 tissues (ear, leaf tip, tassel, leaf base, leaf middle, embryo, root, shoot, anther, and endosperm), under simulated shade (low red/far-red ratio) and far-red (FR) light treatment (Figure 7). The results showed that the majority of the *ZmFAR1* family members were expressed in all tissues; however, their expression levels varied greatly among different tissues. Specifically, certain family members exhibited preferential expression in specific tissues. For instance, the highest expression levels of the *ZmFAR1-1* gene were found in the leaf tip, while the highest expression level of *ZmFAR1-6* was detected in the embryo. *ZmFAR1-15* showed considerably higher expression levels in the leaf tip and leaf middle than in other tissues. Intriguingly, the expression levels of all other *ZmFAR1* genes were highest in the ear and lower in both the anther and endosperm. In addition, all *ZmFAR1s* have different degrees of sensitivity to FR. *ZmFAR1-14* was induced under 1 h FR light treatment, and *ZmFAR1-7*, *ZmFAR1-9*, and *ZmFAR1-15* were induced under 6 h FR light treatment. *ZmFAR1-2* was reduced under 1 h and *ZmFAR1-12* was reduced under 6 h of shade treatment. These findings suggest that various members of the ZmFAR1 family might play diverse roles in separate tissues and suggest a potential role for certain members in ear growth and development and the far-red light response.

### 3.8. Expression Analysis of ZmFAR1 Genes under Far-Red Light

**Figure 8:**
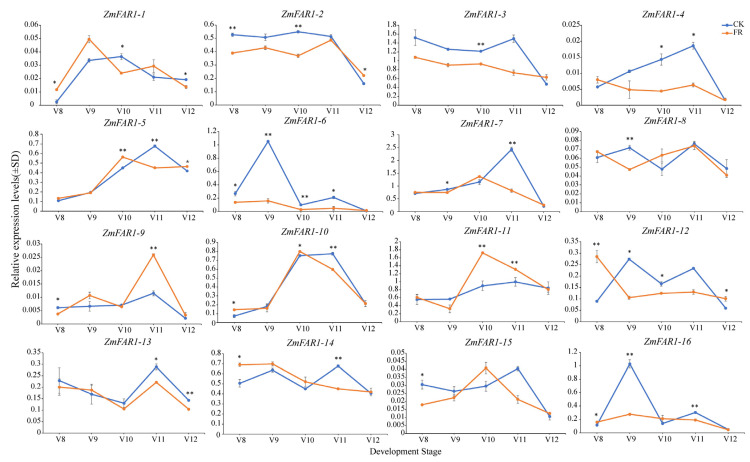
Expression patterns of 16 ZmFAR1 family members in the ear in response to far-red light. Error bars represent the means ± SD from three biological replicates. Statistically significant differences were indicated as follows: ** (*p*-value < 0.01) and * (*p*-value < 0.05), and the indicated *p*-value was determined by Student’s *t*-test. CK (control light condition) and FR (EOD-FR treatment) are represented by blue lines and orange lines, respectively. The abscissa represents the consecutive developmental period; the ordinate represents the qRT-PCR expression levels.

**Figure 9:**
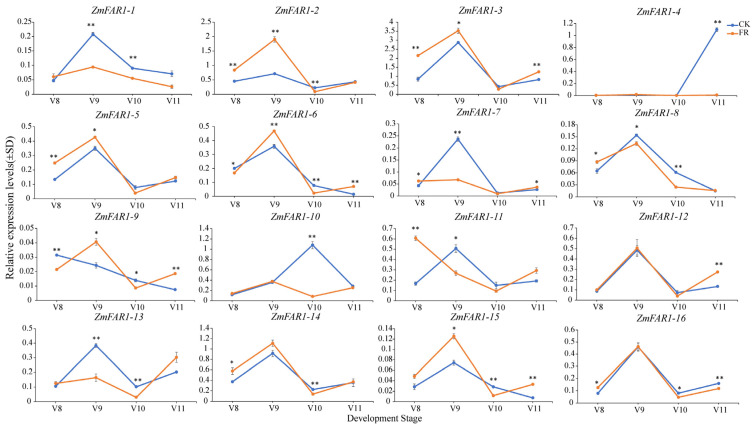
Expression patterns of 16 ZmFAR1 family members in the tassel in response to far-red light. Error bars represent the means ± SDs from three biological replicates. Statistically significant differences were indicated as follows: ** (*p*-value < 0.01) and * (*p*-value < 0.05), and the indicated *p*-value was determined by Student’s *t*-test. CK (control light condition) and FR (EOD-FR treatment) are represented by blue lines and orange lines, respectively. The abscissa represents the consecutive developmental period; the ordinate represents the qRT-PCR expression levels.

Through the transcriptome expression analysis, we found that the *ZmFAR1* genes were highly expressed in maize inflorescence tissues and had a significant response to far-red light treatment. Consequently, we performed a quantitative reverse transcriptase polymerase chain reaction (RT-qPCR) to further verify the 16 *ZmFAR1* genes under far-red light, to determine their expression patterns in the ear and tassel at different developmental stages (V8–V12) (Figure 8 and Figure 9). The results suggest that the peak expression of some *ZmFAR1* genes was significantly influenced by far-red light at various developmental stages and that the expression patterns of certain *ZmFAR1* genes might be limited to a specific period. In the ear, the peak expression of *ZmFAR1-10*, *ZmFAR1-11*, and *ZmFAR1-15* was advanced from V11 to V10, the peak expression of *ZmFAR1-1* was advanced from V10 to V9, and the peak expression of *ZmFAR1-12* was advanced from V9 to V8 (Figure 8).

During the V8–V12 stage in the tassel, the ZmFAR1 family members also showed varied expression patterns, with some being significantly up- or downregulated. We found that *ZmFAR1-1* and *ZmFAR1-12* were significantly upregulated under far-red light treatment during the V8 period, while *ZmFAR1-4*, *ZmFAR1-6*, and *ZmFAR1-7* were significantly downregulated under far-red light during the V11 period. No genes were differentially expressed during the V12 period. Compared to the ear, the expression patterns of the *ZmFAR1* genes were significantly different in the tassel (Figure 9). *ZmFAR1-2*, *ZmFAR1-3*, *ZmFAR1-5*, and *ZmFAR1-11* were significantly upregulated during the V8 period, and *ZmFAR1-2*, *ZmFAR1-9*, and *ZmFAR1-15* were significantly upregulated during the V9 period. Far-red light treatment upregulated *ZmFAR1-6*, *ZmFAR1-9*, *ZmFAR1-12*, and *ZmFAR1-15* during the V11 period, while completely suppressing the expression of *ZmFAR1-4*.

Our findings demonstrated that the expression levels of *ZmFAR1* genes varied significantly between the ear and tassel during the same developmental stage, as well as between developmental periods within the same tissue. In our prior studies, one period in advance of ear development in the treatment group was observed under far-red light [40]. Combined with the analysis of the PCR results, we suggest that six genes with earlier peak expression in the ear (*ZmFAR1-1*, *ZmFAR1-10*, *ZmFAR1-11*, *ZmFAR1-12*, *ZmFAR1-14*, and *ZmFAR1-15*) might have roles in the growth and development process of the ear under far-red light conditions. However, the precise functions of these genes need to be explored further.

### 3.9. Cis-eQTL Analyses Identify the Causal Variations Associated with Gene Expression Variation and Agronomic Traits

**Figure 10:**
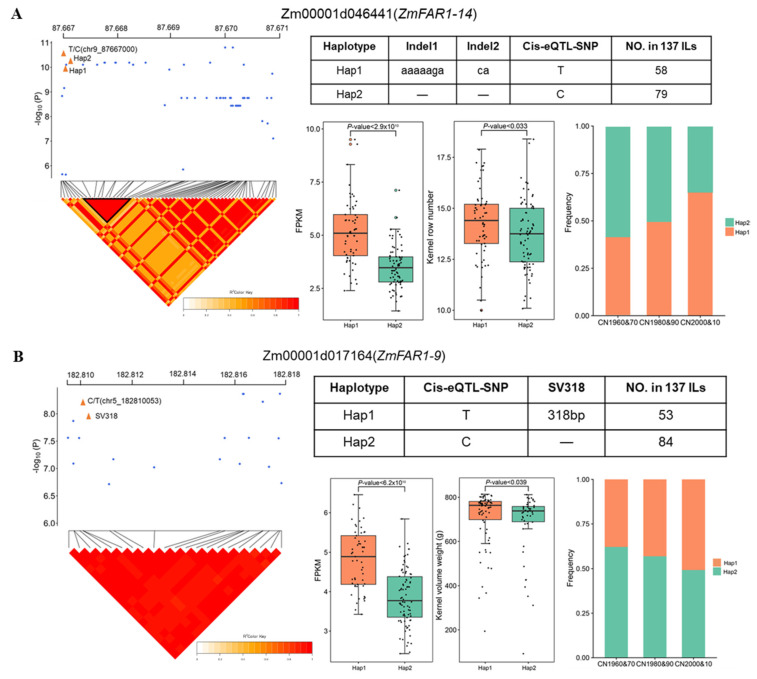
eQTL analyses identify the potential functional variations for expression variation of *ZmFAR1-14* and *ZmFAR1-9*. (**A**) Manhattan plot (upper) and LD heatmap (lower) for eQTLs and candidate association analysis of *ZmFAR1-14*. Two haplotypes of *ZmFAR1-14* based on the two SVs in the 137 ILs (right top). (d) Box plots for *ZmFAR1-14* expression levels and kernel row number for the two haplotypes in the 137 ILs. Bar chart for haplotype frequency changes during modern maize breeding. (**B**) Manhattan plot (upper) and LD heatmap (lower) for eQTLs and candidate association analysis of *ZmFAR1-9*. Two haplotypes of *ZmFAR1-9* based on the two SVs in the 137 ILs (right top). Box plots for *ZmFAR1-9* expression levels and kernel row number for the two haplotypes in the 137 ILs. Bar chart for haplotype frequency changes during modern maize breeding.

To identify the genetic basis of *ZmFAR1* gene expression variation, we performed eQTL analysis based on published RNA-seq data for immature ears and the high-quality single-nucleotide polymorphisms (SNPs) of 137 ILs, using the linear mixed model in the EM-MAX software. We detected a cis-eQTL (B73_V3, chr9_87667000, *p*-value = 2.92 × 10^−11^) associated with the expression level of *ZmFAR1-14* and a cis-eQTL (B73_V3, chr5_182810053, *p*-value = 6.42 × 10^−9^) associated with the expression level of *ZmFAR1-9* (Figure 10). We next attempted to identify the causal variations of several key genes regulating important agronomic traits, taking advantage of the recently released de novo assembled genomes of 14 founder inbred lines (FILs) of maize [41,42,43]. We compared the genic region of *ZmFAR1-14* and 10 kb upstream and downstream sequences in the 14 FILs and found that variations in two indels (indel1(aaaaaga), indel2(ca)) located in the promoter were in high linkage disequilibrium (LD, r^2^ = 0.58, r^2^ = 0.43) with the cis-eQTL (Figure 10). Genotyping analysis of the 137 inbred lines identified two haplotypes (Hap1 and Hap2); the expression level of Hap1 was significantly higher than that in the ILs of Hap1, and the frequency of Hap1 continuously increased during modern maize breeding. We also found that a higher frequency of Hap1 was associated with a higher kernel row number. These results suggest that *ZmFAR1-14* likely plays a role in regulating the kernel row number in maize.

Similarly, by comparing the genic region of *ZmFAR1-9* and 10 kb upstream and downstream sequences in the 14 FILs, we found that variations in a 318 bp SV (SV318, located in the first intron) showed high LD with the lead SNP (r^2^ = 0.61). The SV and cis-eQTL form two haplotypes in the 137 ILs. The expression level and kernel volume weight of Hap1 were significantly higher than those in the ILs of Hap2, and the frequency of Hap1 continuously increased during modern maize breeding. Together, these results suggest that Hap1 of *ZmFAR1-9* likely represents a favorable haplotype under selection for kernel weight during modern maize breeding.

## 4. Discussion

### 4.1. Characterization of the ZmFAR1 Family Members in Maize

*FAR1/FHY3* is a novel transcription factor derived from transposase mutations and has evolved through long-term domestication and adaptation to form the *FAR1/FHY3* transcription factor family [7,44]. Recent studies revealed that *FAR1/FHY3* were crucial positive regulatory components in the *phyA* signaling pathway, primarily activating the transcription of light-induced target genes [7]. In *Arabidopsis*, more than 1000 possible *FHY3* binding genes have been identified, including *ERF4*, *ARC2*, *HEMB1*, and *COP1*, indicating that *FAR1/FHY3* might be involved in the control of several plant growth and development features [6]. However, there have been no reports of a thorough investigation of the *ZmFAR1* transcription factor family in maize. In this study, we identified a total of 16 *ZmFAR1* genes from the maize inbred line B73. The number of *ZmFAR1* genes was greater than that in *Arabidopsis* (14 members) but less than that in *Aarchis hypogaea* (246 members), *Camellia sinensis* (25 members), *Populus trichocarpa* (47 members), *Rosa wichuraiana* ‘*Basye’s Thornless*’ (91 members), and *Rosa chinensis* ‘*Old Blush*’ (50 members) [8,19,44,45]. These findings indicate that the *FAR1/FHY3* gene family expanded throughout the evolution of these species. Despite a recent whole-genome duplication (WGD) event that occurred in maize after species formation, the number of genes belonging to the FAR1/FHY3 family did not vary dramatically. This suggests that the FAR1/FHY3 family is relatively conserved in maize.

### 4.2. Studies on the Structure, Evolutionary Characteristics, and Functions of Maize ZmFAR1 Genes

There were a total of 16 *ZmFAR1* genes that were unevenly distributed across eight chromosomes. The analysis of the physicochemical properties revealed that the majority of the *ZmFAR1* proteins in maize were unstable (with the exception of *ZmFAR1-3* and *ZmFAR1-4*), which was in line with the findings in *Arabidopsis thaliana* [44]. Based on the phylogenetic tree analysis results, all the *ZmFAR1* genes were divided into five subgroups according to the classification of the *FAR1* genes in *Arabidopsis*, with group I including no *ZmFAR1* genes and group V containing the highest number of *ZmFAR1* genes (Figure 1). Based on these findings, it seemed that the *ZmFAR1* genes in group I had been lost during evolution during maize breeding, while the *ZmFAR1* genes in group V might have evolved independently with unique functions. Gene structure analysis revealed that all *ZmFAR1* genes contained the FAR1 domain (Supplementary Figure S1), indicating that the C2H2 zinc finger domain was conserved in maize. Furthermore, the majority of the *ZmFAR1* genes in the same group had similar motifs (Figure 2B), and two FAR1 domains were found in *ZmFAR1-1*, *ZmFAR1-11*, and *ZmFAR1-12*, which was also similar to the results of *Arabidopsis* and poplar [5,8]. Based on the phylogenetic analysis and conserved domain analysis, the *ZmFAR1* genes were shown to be highly conserved at the protein level, and genes within the same subclade might have comparable roles. The significant changes in the number of exons and introns between the *ZmFAR1* genes might be associated with functional differences. Through binding to transcription factors, cis-acting regulatory elements can precisely activate gene transcription and influence transcriptional efficiency, and they have been implicated in several growth and development-related events in plants. Several cis-regulatory elements were found in the promoter regions of the *ZmFAR1* genes, such as the GT-1-motif, GATA-motif, Box-4, and G-box, suggesting that the *ZmFAR1* genes in maize might be involved in light-sensitive responses (Figure 5) [46,47,48]. There are regulatory relationships between the light response and various hormones [14,49]. For example, the *ZmFAR1-9* promoter contained six TGACG motifs and CGTCA motifs, which suggested a possible association with the MeJA signaling pathway [50,51]. By binding directly to the promoter of *ABA-insensitive 5* (*ABI5*) in *Arabidopsis*, FAR1 and FHY3 function as positive regulators of ABA signaling. The *ZmFAR1-10* promoter contained five ABRE motifs and might be related to ABA synthesis and signaling [15,52]. Many types of plant growth and development-related cis-regulatory elements, including the CAT-box, GCN4-motif, and O2-site (seed-specific regulation), were discovered in the promoter regions of several *ZmFAR1* genes, and these cis-elements had a significant role in regulating the expression of meristematic tissue genes. The RY promoter element was involved in controlling gene expression during late embryogenesis and seed development. In the light-induced starch of *Arabidopsis*, *FAR1* and *FHY3* might directly target the starch-debranching enzyme *isoamylase2* (*ISA2*) [11]. In addition, the *FAR1* genes in Arabidopsis could also regulate seed germination [15]. In the present study, RY-element motifs were identified in the promoters of *ZmFAR1-1* and *ZmFAR1-11*, suggesting possible similar functions. Ten and eight *ZmFAR1* genes were predicted to be targeted by miR2275 and miR159, respectively. Previous studies have demonstrated that the production of 24-nt phasiRNAs is mediated by miR2275, which was assoWciated with maize pollen fertility at various temperatures [53,54]. miR159 is very conserved in plants and several light, and stress response cis-elements, such as G-boxes, AREs, and CAAT-boxes, have been found in their promoters and are also involved in male reproductive development, seed development, flowering time, and growth [55,56,57,58]. These results show that miR2275 and miR159 might participate in maize growth and regulation by interacting with the *FAR1* genes, but further results have not been reported.

### 4.3. Functional Variations and Expression Patterns of ZmFAR1 Genes in Maize Ear Development and Phenotypic Variation

The expression patterns of the *ZmFAR1* genes in several organs of the maize inbred line B73 exhibited considerable variances, and the majority of the *ZmFAR1* genes were strongly expressed in the ear, indicating that these genes likely play a significant role in ear growth and development (Figure 7). Previous research had shown that the FAR1 family members *FRS7-FRS12* in *Arabidopsis* could regulate the flowering time and plant growth [37]; among them, *FRS9* was also a specific negative regulator of *PhyB* signaling that could mediate seedling de-etiolation [5]. Because of this, it was hypothesized that members of the same group, such as *ZmFAR1-1*, *ZmFAR1-10*, and *ZmFAR1-14*, could have functional similarities. Importantly, the qRT-PCR results demonstrated that the peak expression of three genes, *ZmFAR1-1*, *ZmFAR1-10*, and *ZmFAR1-14*, was advanced by one stage, which was consistent with the phenotype of ear development in our previous study (Figure 8) [40]. In addition, three expression patterns of the 16 *ZmFAR1* genes were found, with some genes exhibiting phase-advanced, upregulated, or downregulated peak expression under far-red light. Our analysis suggested that certain genes with earlier peak expression in the ear might play roles in growth and development under far-red light conditions, while nondifferentially expressed genes might be nonfunctional. These findings suggest a potential functional redundancy among ZmFAR1 family members, while further research is required to identify the particular activities of these genes. With the recent accumulation of high-density gene data and related high-throughput transcriptome data in maize, eQTL has been used to determine the mutual regulation of genes in the genome [59,60,61]. In this study, our analyses provided new insights into the expression regulation mechanism and phenotypic variation of *ZmFAR1* genes. In combination with eQTL mapping and association studies, we identified several SVs in the promoter of *ZmFAR1-14* as potential functional variations underlying its differential expression and likely regulating the kernel row number. We also found SVs in *ZmFAR1-9* in regulating gene expression and the kernel volume weight (Figure 10). These findings provide novel and important insights into the natural variations in the *ZmFAR1* genes at the transcriptome level and the influence of gene expression on phenotypic variations.

### 4.4. ZmFAR1 Genes’ Role in Maize Yield and Trait Optimization

By uncovering the functional roles of the ZmFAR1 genes and their correlation with maize phenotypic traits such as ear development and the kernel row number, our research offers strategic insights for breeders aiming to enhance yields and environmental resilience. Utilizing genetic variants like SNVs in regulatory regions sheds light on the impact of ZmFAR1 expression on maize diversity, presenting opportunities in marker-assisted selection to foresee and promote desirable traits. This pivotal study not only sets the stage for the advanced functional investigation of the ZmFAR1 gene family but also charts a course for their deliberate application in optimizing agricultural outputs and conserving genetic variation.

## 5. Conclusions

In this study, 16 *ZmFAR1* genes were identified from the maize B73 genome and unevenly distributed over eight chromosomes. The cis-acting regulatory elements of 16 *ZmFAR1* genes were mainly related to the light response, hormone response, stress response, and plant growth and development. RNA-seq analysis showed that most *ZmFAR1* genes showed relatively higher expression levels in the ear and may play a pivotal role in the far-red (FR) light treatment of maize. The qRT-PCR study revealed the spatial and temporal specificity of *ZmFAR1* gene expression under far-red light conditions. Combining our previous phenotypic data, *ZmFAR1-1*, *ZmFAR1-10*, *ZmFAR1-11*, *ZmFAR1-12*, *ZmFAR1-14*, and *ZmFAR1-15* might have roles in ear development under far-red light treatment. Finally, we identified several SVs in *ZmFAR1-14* and *ZmFAR1-9* that contributed to the altered expression patterns and phenotypic differentiation. Overall, our study provided insights into understanding the functions of *ZmFAR1* genes during the light signaling pathway and identified the potential functional variations that may lead to increased yield gains during modern maize breeding.

## Data Availability

Existing datasets are available in a publicly accessible repository. Publicly available datasets were analyzed in this study. These data can be found at http://www.ncbi.nlm.nih.gov/sra/ with the accession numbers of ERR3773807 to ERR3773827.

## Supplementary Materials

The following supporting information can be downloaded at: https://www.mdpi.com/article/10.3390/cimb46010027/s1, Figure S1: Multiple sequence alignment results for the ZmFAR1 family members. The FAR1 domains (motif3-motif9-motif8-motif6) are highlighted in red boxes; Figure S2: The sequence logos of all 15 motifs, where the base size at each point shows the likelihood that the base occurs there; Table S1: The protein sequences of 16 *ZmFAR1* genes in maize B73; Table S2: The cis-acting elements in the promoter regions of ZmFAR1 family members; Table S3: The interactions between miRNAs and putative target *ZmFAR1* genes; Table S4: The primer sequences used in qRT-PCR.
