# Supplementary material for: Genome-Wide Identification and Expression Analyses of the FAR1/FHY3 Gene Family Provide Insight into Inflorescence Development in Maize
Source: Curr Issues Mol Biol. 2024 Jan 2;46(1):430–49. doi: 10.3390/cimb46010027 (PMC10814199; doi:10.3390/cimb46010027)
Supplement: Supplementary file 1 [file cimb-46-00027-s001.zip › cimb-2780978-supplementary.pdf]

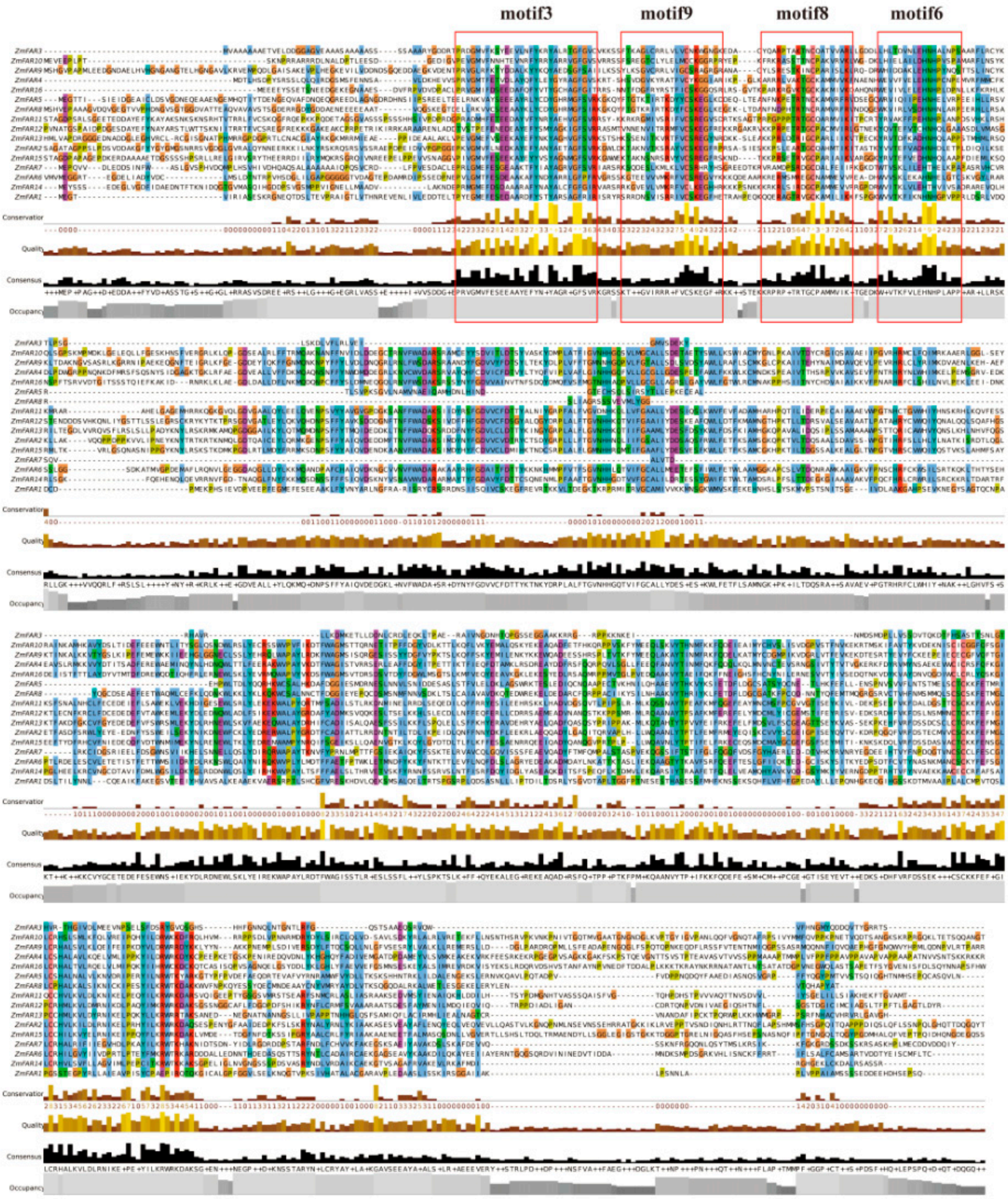

Supplementary Figure S1. Multiple sequence alignment results for the ZmFAR1 family members. The FAR1 domains (motif3-motif9-motif8-motif6) were highlighted in red boxes.

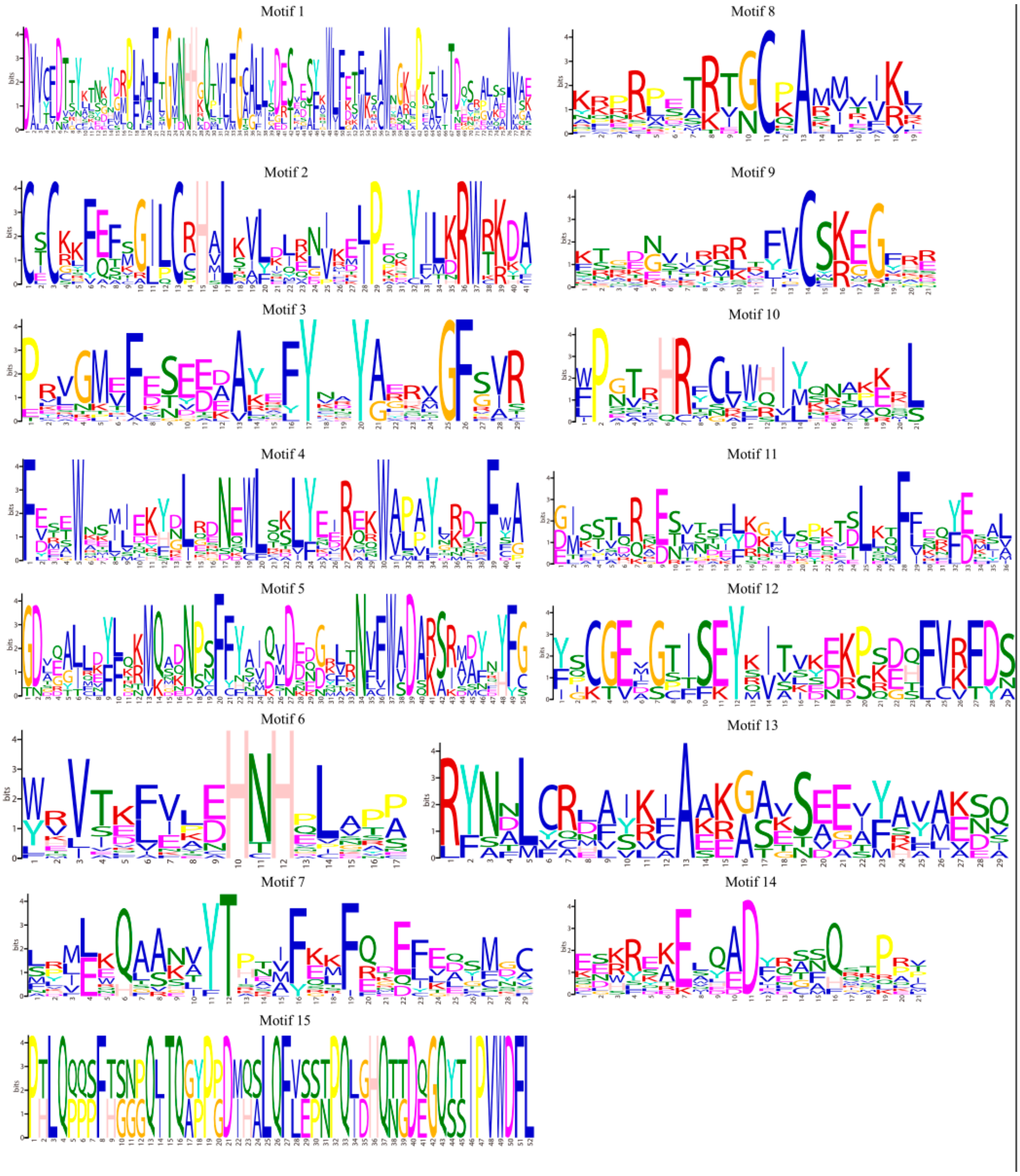

Supplementary Figure S2. The sequence logo of all 15 motifs and the base size at each point showed the likelihood that the base occurred there.

Supplementary Table S1. The protein sequences of 16 *ZmFAR1* genes in maize B73.

Gene Locus ID	Gene Name	Protein Seq
Zm00001d028472	*ZmFAR1-1*	MEGTVIRIASESKRGNEQTDSLTEVPRAIGTLVTHNREVENLIVLEDDTELTPYEGMEFESEDAARDFYSTYARSAGFRIRISRYTRSRRDNSVISRRIVCSKEGFHETRACDGLHPEQKQQERAGTRVGCKAMILIKKFSPGKWVVTKFIKNHNHGPVPPRKLDSRLVDQDCDPMEKPHSIEVDPVEEPFEGMEFESEEAAKLFYVNYARLNGFRARISRYCRSRRDNSIISRQIVCSKEGFREVRTKKVLTDEGKTKRPRMITRVGCKAMIVVKKMNSGKWMVSKFEKEHNHSLSYSKMVPSTSNITSGEIVDLAAKGAHPSEVKNEGYSAGTQCNPADSLTILYNNLCQEAIKFAKEGSVTEEIYHVAVSALKEAAEKVAEVKRSRPTLSHCGFVRESKHDVLQEKSMSALQCSNQVELTRTSPGSRPLQDSASNLLLIPTDILTDSRLYSGVDTAPLTGGFPTNESESTHASESSFMHFKNTKKTSSEKSQHFLVFHFGPEDAYLLLEPQNTCFNQLIHGKEQGIHGSSKDTMVAIPAIPLALCMPVTQSLPGSSTEGPYRLLAAPIEAVPISYCPAEPIRQTQKGICALGPFGGVLSELKNQGTVPKSIVHATALACGARAVPLEDAASLISAIESKIRSGGAIIAKLPSNNLAPLVPPAIAMSSSSEDDEEHDHSEPSQ
Zm00001d034282	*ZmFAR1-2*	MVDRAAARAQAVSSDPPPPALLEPDPRLSQPGAADGQEVASAGATAGPPSLPQASDAEEDALASPAGQPGERCRAMMEVVAKDGAWKVTKLVVEHCHELQVAPGHVAVTVPALGMEFDSVDDAKGFYYGYGEQVGFKARMGSNRRSVGDGEKILQRFLCWKGNYANRSRCKDSDAGKETDEVLEGLSAAAGKRKREPYKTRSRNPGRSTEVIEVEKGVGLGGAGNGLELDNGRRSRRGRSKKAEVEHGEDSVVGFEAEEVAKAVSDADEEEDEGDEDQEAAEQEVEVEVKEQRARGRPRKAVMEDNALQARVLRELGVRALQYNNEERKKILNKYRSKRQSRSVSSRPTKISSRQALAERRKRGNGGRFLSSEEQPLPSERRSKRLKKQNLKMQKAESKEDETMEAEPDPEIDVVPGPGGEPKVGMVFLNEDKAYEFYANYAETAGFSVRKGWLDKTAKNVTKSRAYVCSKEGFRPRSASIESKKPSLEARTGCQAHMTIKITASTKYVVTEFVADHNHDLETPLVDIQILKSEKLLAKVQQPPDPPKVVLIPNEYKNYTRTKRTKNMQLGDTQAICEYLQRMKGENPSFFYAIQVDEDDMFTNVFWADAKSIMDYNYFGDVVCVDTRYCTSDYGRPLLLFTGVNHHNQLIIFGSALIYDDSAQSFRWLFETFKSAMSGKQPKTVLTDQSAALSDAVSSWPGTIHRFSLLHLYLNATKISRDTLQGLETFASDFSRWLYEYEEDNFYSSWEILSEKYNIKDNEWFCKLYEDRERWALPYGRDTFCADIATTLRRDNTNTILTDLIKPEIDLQNFFNNYDKFLEEKRLAEQQADYLGAQITQRVAPLHLLWQAANLYTPTLFEMFRMEYEQISKCVVYSCGEIGPISEYQVTVKDRPQGQFVRFDSTECMVVCSCKKFEFMGLLCCHVLKILDLRNIKELPRHYILKRWRKDAQSESPENYGFAAIDEDPKFSLSKRYNALYRNLYKIAAKASESVEAYAFLENQYEQLVEQVEVLLQAKLHDKSSLSTVLKGNQPNMLNSEVNSSEHRRATGKKIKNVEVRRQQQSLDPNKKKKGRQVLLEPEIEIPLRVEPPTVSNDIQNHLRTTNQFLAPSHMMQAPYVAQQFGLGSLQGFPGMSPFGQIQEPTPLQQPHLQPPSFHSGPQITQAPPPDIQSLQFLSSNPQLGHQTTDQGQYTIPVWDFL
Zm00001d034813	*ZmFAR1-3*	MVAAAAAAETVELDDGGAGVEAAASAAAAASSSSAAARYGDDRTPRDGMVFKSYEEVLNFYKKRYALRTGFGVCVKKSSFTKAGLCRRLVLVCNKWGNGKEDACYQARPTAKTNCQATVVARLLGDDLLHLTDVNLEHNHALNPSAARFLRCYKTLPSGLSKDLVFLRLVEIGMVSDEKYRHAVRLLKDMKETLLDDNLCRDLEQKLTPAERAIVNGDNHTQPGSSEGGAAKKRRGRPPKKNKEINMDSMDPLLVSSDVTQKDTFHSASTTSNLGTHVRTHGIVDLMEEVNPSELSFDSRYGVQSGHSHHFGNNQLNTGNTLRFGQSTSAAEQSRVQWVFHNGMYQDDQVTYGRRTS
Zm00001d003302	*ZmFAR1-4*	MDTLHSDPYSRSSLQLQIRDGSMSFENNSAVLDKHEVVSPRVGMTFETVDLAYQFYLEYGYRAGFGVSKRTSHSVDGVKYRATFVCYKGGIARIKPGLKARRRLVAKTGCKAMMVVKFNASENHWEVVFVELEHNHPCNPEMVRFMMCFKDLPDWQREHRPFNAKTRLNPKIHSGRGRPPNQNKDFMVRSFSQSNYSIDGAGKTGKLRFAEGDVEALLVFFDKMQAQNSNFFYNWDMDDEGRLKNVCWVDARSRVAYQHFCDVICFDTVYLTYQFVIPLVAFLGINHHGQFVLLGCGLLGDESPETFAWLFKKWLKCMNDKSPEAIVTTHSRPVVKAVSEVFPNTRHRYNLWHIMKELPEMSGRVEDKEAVSLRMKKVVYDTITSADFEREWAEMINQYNLHDNQWL

		TTLFEERAKWVPAYVKDTFWAGISTVRRSERLEAFFDGYITPETTIKTFIEQFD<br>TAMKLRSDREAYDDFRSFQQRPQVLSGLLFEEQFANVYTINMFQKFQDQLK<br>QLMNVNCTEVSRNGSIVTYTVTVIGKERKFDYRVMYNSAEKEVWCICRSFQ<br>FKGILCSHALAVLKQELVMLIPPKYILDRWRKDYKCPEEPKETPISQKAAKDT<br>GKGSKPENIREDQVDNLYKHGHQYFADIVEMGATDPDAMEYVLSVMKEAK<br>EKVRKFEESRKEKRPGEGPVSAGKKGAKFSKPSTQEVGNTTSVSTPTEAVAS<br>VTVVSSPPMAAAPTMMAMAPPSAAVAGGMFLVPMHPHPLVFPPFPPAVPPA<br>VAPVAPPAAPATNVVSNTSKKRKKRKGNN
Zm00001d041923	*ZmFAR1-5*	MEGTTISIEIDGEAICLDSVGDNEQEAAENGEMHQTIYTDENGEQVAFDNQE<br>QGREEDLAGNGEEDRDHNSIIPSREELTEELRNKVAYSEEEAYRLYCDYGHR<br>MGFSVRKGKQYYFTGTKTIRTKDYYCSKEGLKDDEQLTEANFNKPETRTNC<br>KAMVRFRVDSEGQWRVIQIIPEHNHELVRPEEIHLLRSVRTLSVPKSGVLNA<br>MVNAEIQAMHDNLHINEDGTECHSQLSIRSYTLLEPKECEALEHPWLTDLYQ<br>QRHKWCSALHKDAFDGGIESMDRNESLNNVLSNIDDESASLSTFVLELDKLA<br>GSWRKTESLEDIQCNQAAPECTVKHNRILQHAAEVYTHKVYKSIETDFLDGC<br>SATSYQAAQCNETLHKFEFLLENSPNVSVVFLNTSTMELSCTCKKFETMRILC<br>SHALNALVLKNVDRIPERYILNRWTKYARKGTYPFPVDEFAEQDRTEVAFVY<br>RNRAMWFVYDLLTKSKSHHNTRKLILDALENGEKSLERNVKQAVLPQTADP<br>VFVDPPNQDQYFAAEDIASNQSVGRPFFYQGYPMTVVSTSQIQGHTNMHSEP<br>QCASQVLN
Zm00001d044646	*ZmFAR1-6*	MVMVMEGGRTEGDELIADYVDCLMSLDTNTRPVHSDGLILGAPGGGGGTV<br>DAGTEPDAMRDIPSSEDPNEPVLGMTFESDEAAKAFYNDYARRLGFPFRVGR<br>SRRSKGTEEVVVMKRFVCSREGVYKKKQTSPDEAARKRERMSMREGCNAM<br>MEVVREADHWVVSKLEKAHNHELGTCSAKVGYLRARSSLGGSDKATMVGP<br>DEMAFLRQNVLGEGGDAQGLLDYLKKMQANDPAFCHAIQVDKNGCVVNV<br>FWADARAKAAYRHFGDAITFDTTYKKNKHMMPFVTFSGVNHHLQTVIFGC<br>ALLMEETEFSFIWLFETWLAAMGGKAPCSLVTDQNRAMKAAIGKVFPNSCH<br>RFCKWSILSRTKQKLTHTYSEHPTLRDELESCVLETETISTFETTWMSIIDRYD<br>LRKNSWLQAIYNIRQKWVPLYLMDTFFAETFPTWKLETMNDFYKKYFNTKT<br>TLEVFLNQFDLSLAGRYEDEAKADMDAYLNKATTKTASLIEKQAAGTYTKA<br>VFSRFQEEFTESLGFIIQKTEDGCISKYSITKYEDPSDTFCVTYNASNKMANCS<br>CKYFEFSGILCRHILGVYIIVDPRTLPTEYFMKRWTRKARDDDALLEDNNNN<br>THDEDASQSTTSRYNTLCADAIRCAEKGAGSEAVYKAAKDILQKAYEEIIAY<br>ERNTGQGSQRDVININEDVTIDDAMNDKSMPDSGRKVHLISNCKFFRRTIFLS<br>ALFCAMSARTVDDTYEISCMFLTC
Zm00001d049493	*ZmFAR1-7*	MEPQVVDLEDDSINFWASLGVSPHVDQMPLHSVHIVDHQAQSALAAAAAAI<br>QPQSVCRDLFPVESDACLEPRLGMEFESGEAAKTFYIAYAGRVGFSVRIARSR<br>KSKCTRVRQSRKSQDESLKMLKLVCSRHRYHSGRESNGEDTKRVRAMDPSR<br>DGCDALFEIIRKGKDTWTVSKLILEHTHELKPAPASRVHCVRSQVALVTDLR<br>KCIDGSRIEELFDSGWNSVIIKHELSNNELLQSLYDIRQRWAPAYTKNVFYPR<br>NLMPTTFGSIEKAIQKYFSSKTELRVAVCQLGQVISSSFEAEVQADYFTMFQM<br>PALSTASPVEKQGSSIFTSTIFGLFQGQFVDSFGYHAERLEDDTVHKYRVNRY<br>EGDEEIHTVYFNPDQGTVNCSCCLFESCGILCRHALRIFIIEGVHDLPKAYILK<br>RWTKHAKNIDTSDNYIDLRGDRDDPSTARFNDLFCHVVKFAKEGSKSAEIYA<br>VAKDSLSKAFDEVVQSSKNFRGQQNLQSYTMSLKRSIKKFGKGRDSSDKSSK<br>RSASKHPLMECDDVDDQIY
Zm00001d016772	*ZmFAR1-8*	MSHVEPAAAGVDQVGEGTVVPHDNGVSGTGGDVATTEAQVAVAVSTSGDE<br>RRGDYGDDAENEEEEEAATVQGSKEGTDELLRKVVCSEEAAYKLYCDYGH<br>RMGFSIRKGKQSYFTGTKRIRTKDYFCSKEGLKEGEKLTDANFNDPHTRTNC<br>RAMVRFRVNDQGEWKVIRLVSDHNHNLARPEERHLLRSARSLIAGRSSSVEV<br>MLYGGYQGCDSEAEFEETWAQMLCEFKLQDNKWLKKLYKLKQKWCSALN<br>NCTFDGGIEYEPQCDSMSNMFNNVSDKLTSLCAIAVAVDKQTEDWREKELD<br>EDARCFQRPPACIIKYSDILNHAAKVYTHRIYKLFETDFLDGCGATKFKELPC<br>QDNNTYQFEMTMQGRGSRVCTVHFNMSMMQLSCSCSKFETMGLLCPHALK<br>ALSIKNICKIPESYILKRWTKDAKKWVFNPKQYESSYQECMNDEAAYCNYV<br>MRYAYDLVTKSQGQDALRKALWETLESGEKELERYLENVTQHAPYAT

Zm00001d017164	*ZmFAR1-9*	MSHGVPAPMLEELAREATLADVSILVDGNDAELHVHGNGAEVHDNGTELH<br>GNGAVLKRVEMPQDLGAISAKEVPLHEGKEVILVDDNDSGQEDDAEGKVDE<br>NTPRVGLRFKTYDDALKYYKQYAEDSGFSAIILKSSYLKSGVCRRLVIGCSRA<br>GRGRANACYLSRESTKINCPARISLKLRQDRWLHIDDAKLEHNHPYNQSTTS<br>LINCYKKLTDAKNGVSASRLKGRRNIPAEKEQGNFTEIGRLKFGEGDDEYIQ<br>KFFGNMQNKNPYFFYLVDLDNQGRLRNLFWSDARSRAANDYFGHDVVYFD<br>TSYLTEKYDLPLVFFTGMNNHGQPVLFGTALLSDLSVDSYAWLLRAFLSCM<br>KGLCPKAIITEHYNAIMDAVQEVLPEVRHRLCLYRIMKDVAENLKEHAEFKT<br>INKALKKVTYGSLKIPEFEMEWKKIIEEHGLGGNECLSSLYEHRQLWAPAYL<br>RDKFWAGMSISQRGESISSYYDGFVYPKTSLKQFFSKYEMILENKYKKEWQA<br>DEESSHRSPLTVTKFYMEEQLAKAYTINMFRKFQDELKATMYCDGMPIKVD<br>GRLVTFEVKECSYMEDGKDTESRTYEVYFCKEEPKVEIECECGFVQFTGILCR<br>HALSVLKLQEIFEIPKDYVLDRWRRDYKKLYYNAKKPNEMPLSDIVERSDYL<br>FTQCSQLLNLGFVSESRYLVALKLLREMERSLLDDGLPARDRQPMLLSFEAD<br>APENGQGLFSPQFSEGVKNSQSAHAKRRGRPLKKVTESTDDTVTQPNKEQDF<br>LRSSFVTENTNMIQGPSSASHLEGPHMGVQGGIDLMDGIPNLSFGNHFGMDI<br>NHQHQVPNHQRMQQNNFIQVQAEPHGFGNQWVYHPMLQDNPVLRTPARR<br>AG
Zm00001d017165	*ZmFAR1-10*	MEVEEPLPTSKNPRRARRRDLNALDPTLEESDGEDIGVPEVGMVFNNHTEVN<br>RFYRRYARRVGFGVSVRRSSFSREGTCLYLELMCCKGGRPRYEPKFRKRASS<br>TTNCPAKVRVKLWGDKLLHIELAILDHNHPVSPAMARFLNSYKQLSGPAKR<br>RLRMGGPGTMPVEESSKMPMDKLGELEQLLFGESKHNSFVERGRLKLQPGD<br>SEALRLFFTRMQAKNANFFNVIDLDDEGCTRNVFWADARSRAMCEYYSDVI<br>TLDTSYVASKYDMPLATFIGVNHHGQSVLMGCALLSDETAETYSWLLKSWI<br>ACMYGNLPKAIVTDYCRGIQSAVAEIIPGVRHRMCLFQIMRKAAERLGGLSE<br>YRAINKAMHKAVYDSLTIDEFEEEWNTLITYSGLQSNDWLRSLYECRSSWVP<br>VFIKDTFWAGMSTTQRNETITPFFDGYVDLKTTLKQFLVKYEMALQSKYEKE<br>AQADFETFHKQRPPVSKFYMEEQLSKVYTHNMFKKFQDEIEAIMYCHVSLIS<br>VDGPVSTFNVKECIFFEDGKRTMSKIFAVTYKVDEKNISCICGGFQFSGILCRH<br>SLSMLKFQLVREIPQHYILDRWKKDFRQLHVMRRPPSDLVPNNRMDRYDYL<br>SIRCLQLVDSAVLSDKYRLALRLVRETEKFLLNSNTHDDTQPRIKSRVPKVNK<br>PNIVTGQTMVGAATGNGNDGLKGPEATAVTQVPQSQKGGAEKGIVPTGYIG<br>VPANLQQFVGNQTAFRPSIVYMVPSGVDPHAFGNVMMPVMYQQMFQVPPK<br>PNETVQDTSANGKSKRPRGQKLTETSQQANGTPASASG
Zm00001d021545	*ZmFAR1-11*	MSTAGDPSRLSGESSPSSSTSSGSSSHSSGAADAAATNLALTAPTSALADDTD<br>ADAPTSPRVGTYFETEDDAYEFYKAYAARLGFVVRKSNKSKNSRHTVTRRL<br>FVCSKQGFRQEPKKPQDETAGSGVASSPSLSLVPAPRCPDSRTGCLASLTIKLI<br>PSANAFRVTDFVADHNHPLASSSPAVSLALLSPSSSHHSIVAVASLPDPRDGP<br>RADMHFETEEDAYVFYNRYAEHVGFSVRRSYKKRKRGMIVSRIFVCSREGV<br>SDRTKQEGGAIVIANGGAGSAGTPRPGPPPTRTGCQARMVIKITPCRTYRVAK<br>FFPEHNHPLANPDSVHKLRSHKMRARAHELGAGEMHRRKQGKGVQLGDVG<br>AALQYLEELQVENPSVYYAVGVGPDGKSAVNFFWADAKSIIDYRSFGDVVC<br>FDTTYALNIYGRPFALFVGVDNHKQLLVFGAALLYDESIQSLKWVFEVFADA<br>MHARHPQTILIDERPECAIAAAEVWPGTNHCTGVWHIYHNSKRHLKQVFESS<br>KSFSNALNHCLFECEDEIEFLSAWEKLVEKHDIGESEWLSRLYLEKEKWALP<br>YQRTMFSADILSTLRKDNMINELRRDLSEQEDILQFFRRYESILEEHRSKKLH<br>ADVDGSQVTLPIPSLRMLKQSSNAYTPEAFKMFQGEFEAYMNCMSFPCGVV<br>GTISEYKIVLDEKPSESFVKFDALDGSTTCSCKKFEAVGIQCCHVLKVLDLKN<br>IKELPEQYILKRWRKDARSVQIGEEPTYGSGSVMRSTSEARFSNMCRLASLIA<br>SRAAKSEDVMSYIESQSNAIQKHLDDILHTSYPDMGNHTVASSSQAISFVGTQ<br>HPDHSTPVVVAQTTNVSDVVLIYSGELILLSIAKHEKFTGVAMT
Zm00001d021799	*ZmFAR1-12*	MPSAPGEKDPQVIPRPATGPPLVQTLTPVNATGSPAIDPRLAQPSWPGHVVLL<br>RPCAAWPPHLPVPLLLPHQNVDALEDVAAADVNPAIDSCDEKMLPKVNMLF<br>DGESDAYEFYNAYAEKVGFFVRRSTLWTTSKNIITRRTFVCSREGFREKKKG<br>AKEAKCPRPETRIGCPASLTIRLTANGKYRLTEFVPNHNHQLATASTVHMLK<br>TKKIRRKARAARENLADDTVSTPEFENEDEAYEFYSMYAGKIGFSVRRASMT<br>VNNENVITRRMFVCSKEGFREKKRGAKRVKKPRPETRTGCPACMVIRLGTN<br>EKYQVTEFVTCHNHQLGAAAASDLVMASGSTENDQDDGFDQADRSPDDSV

		HKQNLIYGSTTLSSLEGRSCKRYKYTKTPRSGDVGATLEYLQKVQHDNPSFF<br>YAVKSDDDGNFTNFFWADSKSIVDFFHFGDVVCFDSGYALQGYDRPLALFT<br>GVNHHKQTVIFGAAIIYDESKEAFQWLLDTFKMAMNGTHPKTLLTDRSVAL<br>SEAVAATLPATAHRYCVWQIYQNALQQLSQAFHGSKTLECNFKRCLFDCED<br>EDEFVTAWKEMLEKYDLEDNQWLADLFSIKEKWALAYGRDAFYADMKSV<br>QQKESLTSELKKHLSLECDLLNFFEQFERLLCDRRSAEMEADVNANQSTKKP<br>PSMRMLRQAANAYTPSAFKMFEREFELYMDCMLYICGEMSTIFEYRISVEDK<br>SRDHFVKFDSLNSMMNCTCKTFEFIGIPCRHMLKVLDMRNIKDLPAQYIMKR<br>WRKDAKSGSSNGGCAFLFDGDPDFSHIKRYNFLCRMFSVAAARAATSDESF<br>AYMENQSNILMDQIEQVIQTRPPDIADLIGANCDRTQNPVDNIVAEGIQSHTN<br>FLSGSTDGICIMCIAGSLTFPFTLGAGTLDYR
Zm00001d022142	*ZmFAR1-13*	MMHMLVAPDRGGGELQPYVAPPAEQELELLRDNADDGLEGHVRCLRCGIS<br>GNATPHMRRGPDGPRTLCNACGIAYRKGKMRRMIEAEPPIDEAALAKLVPE<br>VGMEFVSEEKAYEFYNKYAGHVGFSVRKSTSHKSSENITKVRTFVCSREGYN<br>RDKKSLEAKKPRLDTRIGCPARLIIKVTPECKYRVTDFKADHNHQLAPPSTM<br>HMLRSQRILTELQPGEAELSDDSVVTPTTKATGDLVVRQVSFLRSLSLLPADY<br>KNYLRSKRMKAMQPGDGGAILKYLQTMQMDNPSFFYTMQIDEDDKLTNFF<br>WADQKSRDDFNYFGDVLCLDTTYKINGYGRPLALFLGVNHHKQTIIFGAAM<br>LYDESFDSYKWLFDSFKIAMHGKQPAVALIDQSIPLSSAMAAAWPSTTQRIC<br>AWHVYQNSLKHLNHVFQGSKTFAKDFGKCVFGYEDEDEFVFSWRSMLEKY<br>DLRHNEWLSKVFAEKEQWALAYDRHIFCADIISALQAESFSSILKKFLSPQLE<br>LLSFFKHYERAVDEHRYAELQADFQASQSYPRIPPAKMLKQTAHTYTPVVFE<br>IFRKEFELFMDSVLFSCGEAGTTSEYKVASSEKPKEHFVRFDSSDCSCLCTCR<br>KFEFMGIPCCHMLKVLDYRNIKELPQKYLLKRWRRTAKSANEDNEGNATNA<br>NNGSLLIVPAPPTNHHGLQSFSAMIQFLACIRMHLIEALNAGTCRVNANDAFI<br>PCKTPQRWPLKKHWMGRPPSRFNHACVHRVRLGAVGH
Zm00001d046441	*ZmFAR1-14*	MEYSSSEDEGLVGDFIDAEDNTFTKNIDQGTGVMASQIHGDDPSVGSMPPVI<br>GNELLMAADVLAKNDEPRMGMEFDSDAAARAFYNAYALCFGFGIRVARSR<br>SERRKGVEVLVMKRFVCLKEGHHRKKKPVEPSNKKKRKRLSIRDGCPAMM<br>EVVRRGPDRWVITKLVLEHTHVIVSADRAREVQLHRLSGKFQEHENQLQEV<br>RRNVFGDTNAQGLFNYFKKMQSDNSSFFFSIQVDSKNYVSNAVWVDARAR<br>MAYTYFGDAVYFDTTCSQNENMLPFAAFTGVNHHGDTVVFGCALILDRTES<br>SYGWIFETWLTAMDSRLPFSLTTDEGKGIAAAVAKVFPQCFHRLCRWRILSR<br>CKKRLTDARTRFPGLHEELKRCVNGCDTAVIFDMLWGSILDKYGLRDDNWL<br>QSLYEIRHKWVPAYLTSFFFAELSLTHRVETVSKFYRNNFSSRVSLNTFISRFD<br>QYIDGLYASEAQKDITSFSPEQFLKTDMVLEKQARSIYTRAAFETFQLELVEA<br>MQHYAVKVQDGSYMKYYVERNGDPPTRHTVFYNVAEKKAWCECCRFAFS<br>AILCRHVLSVFLLAGVIMLPEPCITKRWTKKAKSGPELIGLNVGNGSSSPDSV<br>ASRYNDLVRDAIKCAEKGTVSAGAFRVAKEVLRKAFMDIRGHGEKLCKDAL<br>RSASSR
Zm00001d026301	*ZmFAR1-15*	MATPSTAGDPAPAGEPVPTPLQGPAQRRISTSRISHIVRTYLDLSSSRKRRSAP<br>KNQPKAGDKERDAAAAETDGSKAGPSSSHPSRLLRELGIRVSRYTHEERRDII<br>LRYMQKRSGRQIVNRAASKVPSRQALAVRRRRGAGGKFLGKDDAQITDKLE<br>EKAEEEPELPPEVVSNAGGVPIVGMVFESEEKAYEYYVSYAGNMGFSVRKG<br>WWEKTAKNSNRSRVYVCSREGFRSKNDTKKPRSETRVGCPARIAIKVARGG<br>KYRVTEFVEDHNHQLAAPFDIEMLKSQRMLTKVRLGSQNASNIPPGYKNYL<br>RSKSTKDMKPGDLRTLMDYFRRMKSDNPSFYYAIQVDENDKAANVFWADA<br>KSIMDYHYFCDVVCLDMIHKTNDCSRPLALFLGMNHHRQMTIFGAAFLYDE<br>SVESFKWLLETFKSAMCGKHPKTILTDGSSALKEALGLTWPGTVHRSCVWQI<br>YQSTVKSLAHMFSAYEEFTYDFRHCVFNIEDEQDFVDTWNMIMEKYNLREN<br>EWLTKLYEDRENWAMPYNRQIFSGEIKSLLQAENVGTKLKQYLGYDTDLTL<br>FLKFFESSAENRRQEEIQADYQANQGVPRTPLPLLWQAVNLYTPTIFELFRKE<br>CEQSMDCMAYGCGEFGSFSEYMITIKNKSKDQLVRFDSSDASVACTCKKFEN<br>SGLLCCHILKVYELRNIKEIPPQYFLKRWRKDAKLVMDETDGFNFDTKSSIPG<br>RYAALCRLFYRIAAKAAENEETFALMASQSDNLLVGVERTLLSTLSDKSSGH<br>SLTDQLTRMAENDYLLSGGLEGIGSTGKKCEVARRRNGLDTNKRKKAKKGQ<br>PDATDGGPTGRELNIGQASFHSEPSNASNQFIPDQLMQGHYVLGHNFGPGSS

		HNLHDNLNQFDQASSAPTLQQQPFTGNGQLTQGYPGDMHALQFVEPTPQID<br>HQNGDEGQSSIPVWDFL
Zm00001d026485	*ZmFAR1-16*	MEEEEYSSETSNEEDGEKEGNAAESDVFRPVDVDPACLPRVGMIFDSEEDAF<br>QFYVTYGCHAGFGITRRSNNTFDGFRYRSTFICSKGGQSRLRSGVTKPARKR<br>GVKTGCKAKMIVKDAHFQNRWEVIVLELEHNHPLDPNLLKFKRHLKNSPFTI<br>NPPQICESEGQQSNSALVHSSRVVDTGITSSSTQIEFKAKIDRNRKLKLAEGDL<br>DALLDFLNKMQDQNPCFFYSLDMNEQGQLRNVFWSDAKSRSSYNYFGDVV<br>AINVTNFSDQYDMQFVSFMGTNHHAQPVLLGCGLLAGRSLGAYVWLFGTW<br>LRCMNAKPPHSIITNYCHDVAIAIKKVFPNARHRFCLSHILNVLPEKLEEIDNK<br>DEIISTFTTLAYDYVTMTDFDREWQDTIQHFRLERNEWLSKLYEVRMQWAP<br>VYVKDSFWAGMSVTDRSDSVTDYFDGWLMSGTSLKMFVEQYEEAVKGKL<br>EKESYEDLRSAQMRPPMVTGLPVEDQAAKVYTAEIFQKFFNEIGHSFHCNYN<br>ILERNESVVTYIVSEHVDQTNKVDYKVAYDNVQGDIWCLCRLYQSKGILCR<br>HALTVLRQELVLMIPQKYIIHRWCKDCKQTCASISQPVSAGNQELGSYDDLY<br>KLGHLYFAEVVEFGSMNSESKEYALSIMREVRDKVISYEKSLRDQRVDSHVS<br>TANFAYNPVNEDFTDDALPISLSTKGWDLTQGQSKRSRKKKLATPTVLDTLK<br>KKTKRAYNKRRNATANTLNTSATATDGITDGTNVQQNPVNEGWQLASTSAP<br>ETFSYGVENISFDLSQYNNAPSFHWPESSSRSQLH

Supplementary Table S2. The Cis-acting elements in the promoter regions of ZmFAR1 family members.

Gene ID	Cis-acting element	Sequence	Information
ZmFAR1-1	Box 4	ATTAAT	part of a conserved DNA module involved in light responsiveness
ZmFAR1-1	TGACG-motif	TGACG	cis-acting regulatory element involved in the MeJA-responsiveness
ZmFAR1-1	TGACG-motif	TGACG	cis-acting regulatory element involved in the MeJA-responsiveness
ZmFAR1-1	TGA-element	AACGAC	auxin-responsive element
ZmFAR1-1	Sp1	GGGCGG	light responsive element
ZmFAR1-1	Sp1	GGGCGG	light responsive element
ZmFAR1-1	Sp1	GGGCGG	light responsive element
ZmFAR1-1	Sp1	GGGCGG	light responsive element
ZmFAR1-1	ABRE	GACACGTACGT	cis-acting element involved in the abscisic acid responsiveness
ZmFAR1-1	ABRE	TACGGTC	cis-acting element involved in the abscisic acid responsiveness
ZmFAR1-1	TCA-element	CCATCTTTTT	cis-acting element involved in salicylic acid responsiveness
ZmFAR1-1	RY-element	CATGCATG	cis-acting regulatory element involved in seed-specific regulation
ZmFAR1-1	ARE	AAACCA	cis-acting regulatory element essential for the anaerobic induction
ZmFAR1-1	ARE	AAACCA	cis-acting regulatory element essential for the anaerobic induction
ZmFAR1-1	GATA-motif	GATAGGG	part of a light responsive element
ZmFAR1-1	G-box	CACGAC	cis-acting regulatory element involved in light responsiveness
ZmFAR1-1	GC-motif	CCCCCG	enhancer-like element involved in anoxic specific inducibility
ZmFAR1-1	CGTCA-motif	CGTCA	cis-acting regulatory element involved in the MeJA-responsiveness
ZmFAR1-1	CGTCA-motif	CGTCA	cis-acting regulatory element involved in the MeJA-responsiveness
ZmFAR1-1	MBS	CAACTG	MYB binding site involved in drought-inducibility
ZmFAR1-1	MBS	CAACTG	MYB binding site involved in drought-inducibility
ZmFAR1-1	MBS	CAACTG	MYB binding site involved in drought-inducibility

ZmFAR1-1	TCCC-motif	TCTCCCT	part of a light responsive element
ZmFAR1-2	ARE	AAACCA	cis-acting regulatory element essential for the anaerobic induction
ZmFAR1-2	ARE	AAACCA	cis-acting regulatory element essential for the anaerobic induction
ZmFAR1-2	ARE	AAACCA	cis-acting regulatory element essential for the anaerobic induction
ZmFAR1-2	ARE	AAACCA	cis-acting regulatory element essential for the anaerobic induction
ZmFAR1-2	I-box	gGATAAGGTG	part of a light responsive element
ZmFAR1-2	G-box	TACGTG	cis-acting regulatory element involved in light responsiveness
ZmFAR1-2	ABRE	ACGTG	cis-acting element involved in the abscisic acid responsiveness
ZmFAR1-2	TGA-element	AACGAC	auxin-responsive element
ZmFAR1-2	TGA-element	AACGAC	auxin-responsive element
ZmFAR1-2	TGA-element	AACGAC	auxin-responsive element
ZmFAR1-2	Box 4	ATTAAT	part of a conserved DNA module involved in light responsiveness
ZmFAR1-2	GTGGC-motif	CATCGTGTGGC	part of a light responsive element
ZmFAR1-2	GT1-motif	GGTTAA	light responsive element
ZmFAR1-2	GT1-motif	GGTTAA	light responsive element
ZmFAR1-2	GT1-motif	GGTTAAT	light responsive element
ZmFAR1-2	GC-motif	CCCCCG	enhancer-like element involved in anoxic specific inducibility
ZmFAR1-2	O2-site	GATGACATGG	cis-acting regulatory element involved in zein metabolism regulation
ZmFAR1-3	TC-rich repeats	ATTCTCTAAC	cis-acting element involved in defense and stress responsiveness
ZmFAR1-3	TCCC-motif	TCTCCCT	part of a light responsive element
ZmFAR1-3	CAT-box	GCCACT	cis-acting regulatory element related to meristem expression
ZmFAR1-3	CAG-motif	GAAAGGCAGAC	part of a light response element
ZmFAR1-3	O2-site	GATGACATGG	cis-acting regulatory element involved in zein metabolism regulation
ZmFAR1-3	GC-motif	CCCCCG	enhancer-like element involved in anoxic specific inducibility
ZmFAR1-3	GC-motif	CCCCCG	enhancer-like element involved in anoxic specific inducibility

ZmFAR1-3	TCT-motif	TCTTAC	part of a light responsive element
ZmFAR1-3	GCN4_motif	TGAGTCA	cis-regulatory element involved in endosperm expression
ZmFAR1-3	ABRE	ACGTG	cis-acting element involved in the abscisic acid responsiveness
ZmFAR1-3	ABRE	ACGTG	cis-acting element involved in the abscisic acid responsiveness
ZmFAR1-3	ABRE	AACCCGG	cis-acting element involved in the abscisic acid responsiveness
ZmFAR1-3	TGA-element	AACGAC	auxin-responsive element
ZmFAR1-3	GATA-motif	GATAGGA	part of a light responsive element
ZmFAR1-3	GATA-motif	GATAGGA	part of a light responsive element
ZmFAR1-3	GATA-motif	GATAGGA	part of a light responsive element
ZmFAR1-3	GATA-motif	GATAGGA	part of a light responsive element
ZmFAR1-3	G-box	GCCACGTGGA	cis-acting regulatory element involved in light responsiveness
ZmFAR1-3	G-box	TACGTG	cis-acting regulatory element involved in light responsiveness
ZmFAR1-3	G-box	GCCACGTGGA	cis-acting regulatory element involved in light responsiveness
ZmFAR1-3	G-box	TACGTG	cis-acting regulatory element involved in light responsiveness
ZmFAR1-3	G-box	CACGAC	cis-acting regulatory element involved in light responsiveness
ZmFAR1-3	ARE	AAACCA	cis-acting regulatory element essential for the anaerobic induction
ZmFAR1-3	ARE	AAACCA	cis-acting regulatory element essential for the anaerobic induction
ZmFAR1-3	ARE	AAACCA	cis-acting regulatory element essential for the anaerobic induction
ZmFAR1-3	ARE	AAACCA	cis-acting regulatory element essential for the anaerobic induction
ZmFAR1-3	Box 4	ATTAAT	part of a conserved DNA module involved in light responsiveness
ZmFAR1-3	Box 4	ATTAAT	part of a conserved DNA module involved in light responsiveness
ZmFAR1-4	Gap-box	CAAATGAA(A/G)A	part of a light responsive element
ZmFAR1-4	ARE	AAACCA	cis-acting regulatory element essential for the anaerobic induction
ZmFAR1-4	ARE	AAACCA	cis-acting regulatory element essential for the anaerobic induction
ZmFAR1-4	ARE	AAACCA	cis-acting regulatory element essential for the anaerobic induction

ZmFAR1-4	ARE	AAACCA	cis-acting regulatory element essential for the anaerobic induction
ZmFAR1-4	MRE	AACCTAA	MYB binding site involved in light responsiveness
ZmFAR1-4	TCA-element	TCAGAAGAGG	cis-acting element involved in salicylic acid responsiveness
ZmFAR1-4	ABRE	CGTACGTGCA	cis-acting element involved in the abscisic acid responsiveness
ZmFAR1-4	TATC-box	TATCCCA	cis-acting element involved in gibberellin-responsiveness
ZmFAR1-4	TATC-box	TATCCCA	cis-acting element involved in gibberellin-responsiveness
ZmFAR1-4	ATCT-motif	AATCTAATCC	part of a conserved DNA module involved in light responsiveness
ZmFAR1-4	TGACG-motif	TGACG	cis-acting regulatory element involved in the MeJA-responsiveness
ZmFAR1-4	GA-motif	ATAGATAA	part of a light responsive element
ZmFAR1-4	GA-motif	ATAGATAA	part of a light responsive element
ZmFAR1-4	ACE	CTAACGTATT	cis-acting element involved in light responsiveness
ZmFAR1-4	MBS	CAACTG	MYB binding site involved in drought-inducibility
ZmFAR1-4	TCT-motif	TCTTAC	part of a light responsive element
ZmFAR1-4	CGTCA-motif	CGTCA	cis-acting regulatory element involved in the MeJA-responsiveness
ZmFAR1-4	GARE-motif	TCTGTTG	gibberellin-responsive element
ZmFAR1-4	TC-rich repeats	GTTTTCTTAC	cis-acting element involved in defense and stress responsiveness
ZmFAR1-5	LTR	CCGAAA	cis-acting element involved in low-temperature responsiveness
ZmFAR1-5	P-box	CCTTTTG	gibberellin-responsive element
ZmFAR1-5	GT1-motif	GGTTAAT	light responsive element
ZmFAR1-5	ACE	GACACGTATG	cis-acting element involved in light responsiveness
ZmFAR1-5	GA-motif	ATAGATAA	part of a light responsive element
ZmFAR1-5	TCT-motif	TCTTAC	part of a light responsive element
ZmFAR1-5	TCT-motif	TCTTAC	part of a light responsive element
ZmFAR1-5	CGTCA-motif	CGTCA	cis-acting regulatory element involved in the MeJA-responsiveness
ZmFAR1-5	CGTCA-motif	CGTCA	cis-acting regulatory element involved in the MeJA-responsiveness

ZmFAR1-5	GC-motif	CCCCCG	enhancer-like element involved in anoxic specific inducibility
ZmFAR1-5	I-box	AGATAAGG	part of a light responsive element
ZmFAR1-5	GATA-motif	AAGGATAAGG	part of a light responsive element
ZmFAR1-5	G-box	TACGTG	cis-acting regulatory element involved in light responsiveness
ZmFAR1-5	G-box	TAACACGTAG	cis-acting regulatory element involved in light responsiveness
ZmFAR1-5	ARE	AAACCA	cis-acting regulatory element essential for the anaerobic induction
ZmFAR1-5	ARE	AAACCA	cis-acting regulatory element essential for the anaerobic induction
ZmFAR1-5	TCA-element	CCATCTTTTT	cis-acting element involved in salicylic acid responsiveness
ZmFAR1-5	TCA-element	TCAGAAGAGG	cis-acting element involved in salicylic acid responsiveness
ZmFAR1-5	TCA-element	CCATCTTTTT	cis-acting element involved in salicylic acid responsiveness
ZmFAR1-5	ABRE	ACGTG	cis-acting element involved in the abscisic acid responsiveness
ZmFAR1-5	ABRE	CGTACGTGCA	cis-acting element involved in the abscisic acid responsiveness
ZmFAR1-5	Sp1	GGGCGG	light responsive element
ZmFAR1-5	ATCT-motif	AATCTAATCC	part of a conserved DNA module involved in light responsiveness
ZmFAR1-5	TATC-box	TATCCCA	cis-acting element involved in gibberellin-responsiveness
ZmFAR1-5	TGACG-motif	TGACG	cis-acting regulatory element involved in the MeJA-responsiveness
ZmFAR1-5	TGACG-motif	TGACG	cis-acting regulatory element involved in the MeJA-responsiveness
ZmFAR1-6	TCT-motif	TCTTAC	part of a light responsive element
ZmFAR1-6	ACE	CTAACGTATT	cis-acting element involved in light responsiveness
ZmFAR1-6	MBS	CAACTG	MYB binding site involved in drought-inducibility
ZmFAR1-6	MBS	CAACTG	MYB binding site involved in drought-inducibility
ZmFAR1-6	MBS	CAACTG	MYB binding site involved in drought-inducibility
ZmFAR1-6	MBS	CAACTG	MYB binding site involved in drought-inducibility
ZmFAR1-6	MBS	CAACTG	MYB binding site involved in drought-inducibility
ZmFAR1-6	GC-motif	CCCCCG	enhancer-like element involved in anoxic specific inducibility

ZmFAR1-6	GARE-motif	TCTGTTG	gibberellin-responsive element
ZmFAR1-6	LTR	CCGAAA	cis-acting element involved in low-temperature responsiveness
ZmFAR1-6	GT1-motif	GGTTAA	light responsive element
ZmFAR1-6	GT1-motif	GGTTAA	light responsive element
ZmFAR1-6	GT1-motif	GGTTAA	light responsive element
ZmFAR1-6	Box 4	ATTAAT	part of a conserved DNA module involved in light responsiveness
ZmFAR1-6	Box 4	ATTAAT	part of a conserved DNA module involved in light responsiveness
ZmFAR1-6	Box 4	ATTAAT	part of a conserved DNA module involved in light responsiveness
ZmFAR1-6	G-Box	CACGTG	cis-acting regulatory element involved in light responsiveness
ZmFAR1-6	chs-CMA1a	TTACTTAA	part of a light responsive element
ZmFAR1-6	G-box	CACGTG	cis-acting regulatory element involved in light responsiveness
ZmFAR1-6	ABRE	CGCACGTGTC	cis-acting element involved in the abscisic acid responsiveness
ZmFAR1-6	ABRE	CACGTG	cis-acting element involved in the abscisic acid responsiveness
ZmFAR1-6	ABRE	ACGTG	cis-acting element involved in the abscisic acid responsiveness
ZmFAR1-6	Sp1	GGGCGG	light responsive element
ZmFAR1-6	ATCT-motif	AATCTAATCC	part of a conserved DNA module involved in light responsiveness
ZmFAR1-6	ATCT-motif	AATCTAATCC	part of a conserved DNA module involved in light responsiveness
ZmFAR1-7	GT1-motif	GGTTAA	light responsive element
ZmFAR1-7	CGTCA-motif	CGTCA	cis-acting regulatory element involved in the MeJA-responsiveness
ZmFAR1-7	CGTCA-motif	CGTCA	cis-acting regulatory element involved in the MeJA-responsiveness
ZmFAR1-7	O2-site	GATGATGTGG	cis-acting regulatory element involved in zein metabolism regulation
ZmFAR1-7	I-box	cGATAAGGCG	part of a light responsive element
ZmFAR1-7	ARE	AAACCA	cis-acting regulatory element essential for the anaerobic induction
ZmFAR1-7	ARE	AAACCA	cis-acting regulatory element essential for the anaerobic induction
ZmFAR1-7	TCA-element	CCATCTTTTT	cis-acting element involved in salicylic acid responsiveness

ZmFAR1-7	TCA-element	CCATCTTTTT	cis-acting element involved in salicylic acid responsiveness
ZmFAR1-7	TGACG-motif	TGACG	cis-acting regulatory element involved in the MeJA-responsiveness
ZmFAR1-7	TGACG-motif	TGACG	cis-acting regulatory element involved in the MeJA-responsiveness
ZmFAR1-7	ABRE	GCCGCGTGGC	cis-acting element involved in the abscisic acid responsiveness
ZmFAR1-7	TGA-element	AACGAC	auxin-responsive element
ZmFAR1-7	Box 4	ATTAAT	part of a conserved DNA module involved in light responsiveness
ZmFAR1-8	GATA-motif	GATAGGA	part of a light responsive element
ZmFAR1-8	ARE	AAACCA	cis-acting regulatory element essential for the anaerobic induction
ZmFAR1-8	ARE	AAACCA	cis-acting regulatory element essential for the anaerobic induction
ZmFAR1-8	ARE	AAACCA	cis-acting regulatory element essential for the anaerobic induction
ZmFAR1-8	TGACG-motif	TGACG	cis-acting regulatory element involved in the MeJA-responsiveness
ZmFAR1-8	TGACG-motif	TGACG	cis-acting regulatory element involved in the MeJA-responsiveness
ZmFAR1-8	Sp1	GGGCGG	light responsive element
ZmFAR1-8	Box 4	ATTAAT	part of a conserved DNA module involved in light responsiveness
ZmFAR1-8	Gap-box	CAAATGAA(A/G)A	part of a light responsive element
ZmFAR1-8	TCCC-motif	TCTCCCT	part of a light responsive element
ZmFAR1-8	CAT-box	GCCACT	cis-acting regulatory element related to meristem expression
ZmFAR1-8	CAT-box	GCCACT	cis-acting regulatory element related to meristem expression
ZmFAR1-8	GTGGC-motif	GATTCTGTGGC	part of a light responsive element
ZmFAR1-8	GT1-motif	GGTTAA	light responsive element
ZmFAR1-8	TC-rich repeats	GTTTTCTTAC	cis-acting element involved in defense and stress responsiveness
ZmFAR1-8	CGTCA-motif	CGTCA	cis-acting regulatory element involved in the MeJA-responsiveness
ZmFAR1-8	CGTCA-motif	CGTCA	cis-acting regulatory element involved in the MeJA-responsiveness
ZmFAR1-8	O2-site	GATGATGTGG	cis-acting regulatory element involved in zein metabolism regulation
ZmFAR1-9	TGACG-motif	TGACG	cis-acting regulatory element involved in the MeJA-responsiveness

ZmFAR1-9	TGACG-motif	TGACG	cis-acting regulatory element involved in the MeJA-responsiveness
ZmFAR1-9	TGACG-motif	TGACG	cis-acting regulatory element involved in the MeJA-responsiveness
ZmFAR1-9	TGACG-motif	TGACG	cis-acting regulatory element involved in the MeJA-responsiveness
ZmFAR1-9	TGACG-motif	TGACG	cis-acting regulatory element involved in the MeJA-responsiveness
ZmFAR1-9	TGACG-motif	TGACG	cis-acting regulatory element involved in the MeJA-responsiveness
ZmFAR1-9	Sp1	GGGCGG	light responsive element
ZmFAR1-9	ABRE	ACGTG	cis-acting element involved in the abscisic acid responsiveness
ZmFAR1-9	ABRE	ACGTG	cis-acting element involved in the abscisic acid responsiveness
ZmFAR1-9	TGA-element	AACGAC	auxin-responsive element
ZmFAR1-9	G-box	TACGTG	cis-acting regulatory element involved in light responsiveness
ZmFAR1-9	G-box	CACGTC	cis-acting regulatory element involved in light responsiveness
ZmFAR1-9	G-box	TAACACGTAG	cis-acting regulatory element involved in light responsiveness
ZmFAR1-9	chs-CMA1a	TTACTTAA	part of a light responsive element
ZmFAR1-9	ARE	AAACCA	cis-acting regulatory element essential for the anaerobic induction
ZmFAR1-9	ARE	AAACCA	cis-acting regulatory element essential for the anaerobic induction
ZmFAR1-9	P-box	CCTTTTG	gibberellin-responsive element
ZmFAR1-9	TCCC-motif	TCTCCCT	part of a light responsive element
ZmFAR1-9	CAG-motif	GAAAGGCAGAC	part of a light response element
ZmFAR1-9	O2-site	GATGATGTGG	cis-acting regulatory element involved in zein metabolism regulation
ZmFAR1-9	GC-motif	CCCCCG	enhancer-like element involved in anoxic specific inducibility
ZmFAR1-9	CGTCA-motif	CGTCA	cis-acting regulatory element involved in the MeJA-responsiveness
ZmFAR1-9	CGTCA-motif	CGTCA	cis-acting regulatory element involved in the MeJA-responsiveness
ZmFAR1-9	CGTCA-motif	CGTCA	cis-acting regulatory element involved in the MeJA-responsiveness
ZmFAR1-9	CGTCA-motif	CGTCA	cis-acting regulatory element involved in the MeJA-responsiveness
ZmFAR1-9	CGTCA-motif	CGTCA	cis-acting regulatory element involved in the MeJA-responsiveness

ZmFAR1-9	CGTCA-motif	CGTCA	cis-acting regulatory element involved in the MeJA-responsiveness
ZmFAR1-9	TCT-motif	TCTTAC	part of a light responsive element
ZmFAR1-10	TCA-element	CCATCTTTTT	cis-acting element involved in salicylic acid responsiveness
ZmFAR1-10	TCA-element	CCATCTTTTT	cis-acting element involved in salicylic acid responsiveness
ZmFAR1-10	TGACG-motif	TGACG	cis-acting regulatory element involved in the MeJA-responsiveness
ZmFAR1-10	TGACG-motif	TGACG	cis-acting regulatory element involved in the MeJA-responsiveness
ZmFAR1-10	TGACG-motif	TGACG	cis-acting regulatory element involved in the MeJA-responsiveness
ZmFAR1-10	Sp1	GGGCGG	light responsive element
ZmFAR1-10	ABRE	ACGTG	cis-acting element involved in the abscisic acid responsiveness
ZmFAR1-10	ABRE	CACGTG	cis-acting element involved in the abscisic acid responsiveness
ZmFAR1-10	ABRE	ACGTG	cis-acting element involved in the abscisic acid responsiveness
ZmFAR1-10	ABRE	ACGTG	cis-acting element involved in the abscisic acid responsiveness
ZmFAR1-10	ABRE	AACCCGG	cis-acting element involved in the abscisic acid responsiveness
ZmFAR1-10	G-box	CCACGTAA	cis-acting regulatory element involved in light responsiveness
ZmFAR1-10	G-box	TACGTG	cis-acting regulatory element involved in light responsiveness
ZmFAR1-10	G-box	CACGTG	cis-acting regulatory element involved in light responsiveness
ZmFAR1-10	G-box	CACGAC	cis-acting regulatory element involved in light responsiveness
ZmFAR1-10	G-Box	CACGTG	cis-acting regulatory element involved in light responsiveness
ZmFAR1-10	G-Box	CACGTT	cis-acting regulatory element involved in light responsiveness
ZmFAR1-10	ARE	AAACCA	cis-acting regulatory element essential for the anaerobic induction
ZmFAR1-10	LTR	CCGAAA	cis-acting element involved in low-temperature responsiveness
ZmFAR1-10	LTR	CCGAAA	cis-acting element involved in low-temperature responsiveness
ZmFAR1-10	LTR	CCGAAA	cis-acting element involved in low-temperature responsiveness
ZmFAR1-10	O2-site	GATGACATGG	cis-acting regulatory element involved in zein metabolism regulation
ZmFAR1-10	ACE	GACACGTATG	cis-acting element involved in light responsiveness

ZmFAR1-10	MBS	CAACTG	MYB binding site involved in drought-inducibility
ZmFAR1-10	CGTCA-motif	CGTCA	cis-acting regulatory element involved in the MeJA-responsiveness
ZmFAR1-10	CGTCA-motif	CGTCA	cis-acting regulatory element involved in the MeJA-responsiveness
ZmFAR1-10	CGTCA-motif	CGTCA	cis-acting regulatory element involved in the MeJA-responsiveness
ZmFAR1-11	CGTCA-motif	CGTCA	cis-acting regulatory element involved in the MeJA-responsiveness
ZmFAR1-11	CGTCA-motif	CGTCA	cis-acting regulatory element involved in the MeJA-responsiveness
ZmFAR1-11	MBS	CAACTG	MYB binding site involved in drought-inducibility
ZmFAR1-11	GC-motif	CCCCCG	enhancer-like element involved in anoxic specific inducibility
ZmFAR1-11	GARE-motif	TCTGTTG	gibberellin-responsive element
ZmFAR1-11	CAT-box	GCCACT	cis-acting regulatory element related to meristem expression
ZmFAR1-11	TCCC-motif	TCTCCCT	part of a light responsive element
ZmFAR1-11	GT1-motif	GGTTAAT	light responsive element
ZmFAR1-11	GT1-motif	GGTTAA	light responsive element
ZmFAR1-11	GT1-motif	GGTTAA	light responsive element
ZmFAR1-11	GT1-motif	GGTTAAT	light responsive element
ZmFAR1-11	GT1-motif	GGTTAA	light responsive element
ZmFAR1-11	P-box	CCTTTTG	gibberellin-responsive element
ZmFAR1-11	ARE	AAACCA	cis-acting regulatory element essential for the anaerobic induction
ZmFAR1-11	ARE	AAACCA	cis-acting regulatory element essential for the anaerobic induction
ZmFAR1-11	G-box	TACGTG	cis-acting regulatory element involved in light responsiveness
ZmFAR1-11	G-box	CACGTC	cis-acting regulatory element involved in light responsiveness
ZmFAR1-11	TGACG-motif	TGACG	cis-acting regulatory element involved in the MeJA-responsiveness
ZmFAR1-11	TGACG-motif	TGACG	cis-acting regulatory element involved in the MeJA-responsiveness
ZmFAR1-11	TATC-box	TATCCCA	cis-acting element involved in gibberellin-responsiveness
ZmFAR1-11	ABRE	ACGTG	cis-acting element involved in the abscisic acid responsiveness

ZmFAR1-11	ABRE	ACGTG	cis-acting element involved in the abscisic acid responsiveness
ZmFAR1-11	TCA-element	TCAGAAGAGG	cis-acting element involved in salicylic acid responsiveness
ZmFAR1-11	RY-element	CATGCATG	cis-acting regulatory element involved in seed-specific regulation
ZmFAR1-12	LTR	CCGAAA	cis-acting element involved in low-temperature responsiveness
ZmFAR1-12	GTGGC-motif	CAGCGTGTGGC	part of a light responsive element
ZmFAR1-12	GC-motif	CCCCCG	enhancer-like element involved in anoxic specific inducibility
ZmFAR1-12	TCT-motif	TCTTAC	part of a light responsive element
ZmFAR1-12	MBS	CAACTG	MYB binding site involved in drought-inducibility
ZmFAR1-12	GCN4_motif	TGAGTCA	cis-regulatory element involved in endosperm expression
ZmFAR1-12	ARE	AAACCA	cis-acting regulatory element essential for the anaerobic induction
ZmFAR1-12	ARE	AAACCA	cis-acting regulatory element essential for the anaerobic induction
ZmFAR1-12	ARE	AAACCA	cis-acting regulatory element essential for the anaerobic induction
ZmFAR1-12	ARE	AAACCA	cis-acting regulatory element essential for the anaerobic induction
ZmFAR1-12	ARE	AAACCA	cis-acting regulatory element essential for the anaerobic induction
ZmFAR1-12	chs-CMA1a	TTACTTAA	part of a light responsive element
ZmFAR1-12	G-box	CACGAC	cis-acting regulatory element involved in light responsiveness
ZmFAR1-12	G-box	TACGTG	cis-acting regulatory element involved in light responsiveness
ZmFAR1-12	AuxRR-core	GGTCCAT	cis-acting regulatory element involved in auxin responsiveness
ZmFAR1-12	AuxRR-core	GGTCCAT	cis-acting regulatory element involved in auxin responsiveness
ZmFAR1-12	CAT-box	GCCACT	cis-acting regulatory element related to meristem expression
ZmFAR1-12	CAT-box	GCCACT	cis-acting regulatory element related to meristem expression
ZmFAR1-12	CGTCA-motif	CGTCA	cis-acting regulatory element involved in the MeJA-responsiveness
ZmFAR1-12	CGTCA-motif	CGTCA	cis-acting regulatory element involved in the MeJA-responsiveness
ZmFAR1-12	CGTCA-motif	CGTCA	cis-acting regulatory element involved in the MeJA-responsiveness
ZmFAR1-12	GA-motif	ATAGATAA	part of a light responsive element

ZmFAR1-12	ABRE	ACGTG	cis-acting element involved in the abscisic acid responsiveness
ZmFAR1-12	Sp1	GGGCGG	light responsive element
ZmFAR1-12	Sp1	GGGCGG	light responsive element
ZmFAR1-12	Sp1	GGGCGG	light responsive element
ZmFAR1-12	TGACG-motif	TGACG	cis-acting regulatory element involved in the MeJA-responsiveness
ZmFAR1-12	TGACG-motif	TGACG	cis-acting regulatory element involved in the MeJA-responsiveness
ZmFAR1-12	TGACG-motif	TGACG	cis-acting regulatory element involved in the MeJA-responsiveness
ZmFAR1-12	MRE	AACCTAA	MYB binding site involved in light responsiveness
ZmFAR1-12	Box 4	ATTAAT	part of a conserved DNA module involved in light responsiveness
ZmFAR1-13	GARE-motif	TCTGTTG	gibberellin-responsive element
ZmFAR1-13	GARE-motif	TCTGTTG	gibberellin-responsive element
ZmFAR1-13	GT1-motif	GGTTAA	light responsive element
ZmFAR1-13	TC-rich repeats	GTTTTCTTAC	cis-acting element involved in defense and stress responsiveness
ZmFAR1-13	TCCC-motif	TCTCCCT	part of a light responsive element
ZmFAR1-13	CAT-box	GCCACT	cis-acting regulatory element related to meristem expression
ZmFAR1-13	CAT-box	GCCACT	cis-acting regulatory element related to meristem expression
ZmFAR1-13	CAT-box	GCCACT	cis-acting regulatory element related to meristem expression
ZmFAR1-13	AE-box	AGAAACAA	part of a module for light response
ZmFAR1-13	TGA-element	AACGAC	auxin-responsive element
ZmFAR1-13	TGA-element	AACGAC	auxin-responsive element
ZmFAR1-13	ABRE	ACGTG	cis-acting element involved in the abscisic acid responsiveness
ZmFAR1-13	ABRE	ACGTG	cis-acting element involved in the abscisic acid responsiveness
ZmFAR1-13	ABRE	ACGTG	cis-acting element involved in the abscisic acid responsiveness
ZmFAR1-13	GATA-motif	AAGGATAAGG	part of a light responsive element
ZmFAR1-13	G-box	TACGTG	cis-acting regulatory element involved in light responsiveness

ZmFAR1-13	G-box	TAACACGTAG	cis-acting regulatory element involved in light responsiveness
ZmFAR1-13	G-box	TACGTG	cis-acting regulatory element involved in light responsiveness
ZmFAR1-13	I-box	GTATAAGGCC	part of a light responsive element
ZmFAR1-13	ARE	AAACCA	cis-acting regulatory element essential for the anaerobic induction
ZmFAR1-13	G-Box	CACGTT	cis-acting regulatory element involved in light responsiveness
ZmFAR1-14	GC-motif	CCCCCG	enhancer-like element involved in anoxic specific inducibility
ZmFAR1-14	CGTCA-motif	CGTCA	cis-acting regulatory element involved in the MeJA-responsiveness
ZmFAR1-14	CGTCA-motif	CGTCA	cis-acting regulatory element involved in the MeJA-responsiveness
ZmFAR1-14	GT1-motif	GGTTAA	light responsive element
ZmFAR1-14	CAT-box	GCCACT	cis-acting regulatory element related to meristem expression
ZmFAR1-14	CAT-box	GCCACT	cis-acting regulatory element related to meristem expression
ZmFAR1-14	CAT-box	GCCACT	cis-acting regulatory element related to meristem expression
ZmFAR1-14	MRE	AACCTAA	MYB binding site involved in light responsiveness
ZmFAR1-14	ATCT-motif	AATCTAATCC	part of a conserved DNA module involved in light responsiveness
ZmFAR1-14	TGACG-motif	TGACG	cis-acting regulatory element involved in the MeJA-responsiveness
ZmFAR1-14	TGACG-motif	TGACG	cis-acting regulatory element involved in the MeJA-responsiveness
ZmFAR1-14	ABRE	GCAACGTGTC	cis-acting element involved in the abscisic acid responsiveness
ZmFAR1-14	Sp1	GGGCGG	light responsive element
ZmFAR1-14	Sp1	GGGCGG	light responsive element
ZmFAR1-14	Sp1	GGGCGG	light responsive element
ZmFAR1-14	Sp1	GGGCGG	light responsive element
ZmFAR1-14	Sp1	GGGCGG	light responsive element
ZmFAR1-14	GATA-motif	AAGGATAAGG	part of a light responsive element
ZmFAR1-14	GATA-motif	AAGATAAGATT	part of a light responsive element
ZmFAR1-14	G-box	CACGAC	cis-acting regulatory element involved in light responsiveness

ZmFAR1-14	ARE	AAACCA	cis-acting regulatory element essential for the anaerobic induction
ZmFAR1-15	TGACG-motif	TGACG	cis-acting regulatory element involved in the MeJA-responsiveness
ZmFAR1-15	ABRE	ACGTG	cis-acting element involved in the abscisic acid responsiveness
ZmFAR1-15	ABRE	ACGTG	cis-acting element involved in the abscisic acid responsiveness
ZmFAR1-15	AE-box	AGAAACAA	part of a module for light response
ZmFAR1-15	AE-box	AGAAACAA	part of a module for light response
ZmFAR1-15	AE-box	AGAAACAA	part of a module for light response
ZmFAR1-15	Box 4	ATTAAT	part of a conserved DNA module involved in light responsiveness
ZmFAR1-15	CAT-box	GCCACT	cis-acting regulatory element related to meristem expression
ZmFAR1-15	GARE-motif	TCTGTTG	gibberellin-responsive element
ZmFAR1-15	CGTCA-motif	CGTCA	cis-acting regulatory element involved in the MeJA-responsiveness
ZmFAR1-15	TATC-box	TATCCCA	cis-acting element involved in gibberellin-responsiveness
ZmFAR1-15	TGA-element	AACGAC	auxin-responsive element
ZmFAR1-15	GATA-motif	GATAGGG	part of a light responsive element
ZmFAR1-15	G-box	TACGTG	cis-acting regulatory element involved in light responsiveness
ZmFAR1-15	G-box	CCACGTAA	cis-acting regulatory element involved in light responsiveness
ZmFAR1-15	G-box	TACGTG	cis-acting regulatory element involved in light responsiveness
ZmFAR1-15	chs-CMA1a	TTACTTAA	part of a light responsive element
ZmFAR1-15	I-box	gGATAAGGTG	part of a light responsive element
ZmFAR1-15	ARE	AAACCA	cis-acting regulatory element essential for the anaerobic induction
ZmFAR1-15	GT1-motif	GGTTAA	light responsive element
ZmFAR1-15	LTR	CCGAAA	cis-acting element involved in low-temperature responsiveness
ZmFAR1-15	O2-site	GATGA(C/T)(A/G)TG(A/G)	cis-acting regulatory element involved in zein metabolism regulation
ZmFAR1-15	ACE	CTAACGTATT	cis-acting element involved in light responsiveness
ZmFAR1-15	MBS	CAACTG	MYB binding site involved in drought-inducibility

ZmFAR1-15	MBS	CAACTG	MYB binding site involved in drought-inducibility
ZmFAR1-15	MBS	CAACTG	MYB binding site involved in drought-inducibility
ZmFAR1-16	ACE	GACACGTATG	cis-acting element involved in light responsiveness
ZmFAR1-16	MBS	CAACTG	MYB binding site involved in drought-inducibility
ZmFAR1-16	MBS	CAACTG	MYB binding site involved in drought-inducibility
ZmFAR1-16	GC-motif	CCCCCG	enhancer-like element involved in anoxic specific inducibility
ZmFAR1-16	TCCC-motif	TCTCCCT	part of a light responsive element
ZmFAR1-16	GT1-motif	GGTTAAT	light responsive element
ZmFAR1-16	GT1-motif	GGTTAA	light responsive element
ZmFAR1-16	LTR	CCGAAA	cis-acting element involved in low-temperature responsiveness
ZmFAR1-16	GATA-motif	GATAGGA	part of a light responsive element
ZmFAR1-16	G-box	TAACACGTAG	cis-acting regulatory element involved in light responsiveness
ZmFAR1-16	G-box	TACGTG	cis-acting regulatory element involved in light responsiveness
ZmFAR1-16	G-box	CAGACGTGGCA	cis-acting regulatory element involved in light responsiveness
ZmFAR1-16	G-box	CACGTC	cis-acting regulatory element involved in light responsiveness
ZmFAR1-16	ARE	AAACCA	cis-acting regulatory element essential for the anaerobic induction
ZmFAR1-16	GCN4_motif	TGAGTCA	cis-regulatory element involved in endosperm expression
ZmFAR1-16	CGTCA-motif	CGTCA	cis-acting regulatory element involved in the MeJA-responsiveness
ZmFAR1-16	GARE-motif	TCTGTTG	gibberellin-responsive element
ZmFAR1-16	CAT-box	GCCACT	cis-acting regulatory element related to meristem expression
ZmFAR1-16	AuxRR-core	GGTCCAT	cis-acting regulatory element involved in auxin responsiveness
ZmFAR1-16	Box 4	ATTAAT	part of a conserved DNA module involved in light responsiveness
ZmFAR1-16	Box 4	ATTAAT	part of a conserved DNA module involved in light responsiveness
ZmFAR1-16	TGACG-motif	TGACG	cis-acting regulatory element involved in the MeJA-responsiveness
ZmFAR1-16	Sp1	GGGCGG	light responsive element

ZmFAR1-16	ABRE	ACGTG	cis-acting element involved in the abscisic acid responsiveness
ZmFAR1-16	ABRE	ACGTG	cis-acting element involved in the abscisic acid responsiveness

Supplementary Table S3. The interactions between miRNAs and putative target *ZmFAR1* genes

miRNA_Acc.	Target_Acc.	Expectation	UPE$	miRNA_start	miRNA_end	Target_start	Target_end	miRNA_aligned_fragment	alignment	Target_aligned_fragment	Inhibition	Target_Desc.	Multiplicity
miR156a-5p	*ZmFAR1-5*	3	-1	1	20	2715	2734	UGACAGAAGAGAGUGAGCAC	::::.:::::.:: :: :.:	UUGCUUACUCUUUUCUUUUA	Cleavage	reverse complement	1
miR156b-5p	*ZmFAR1-5*	3	-1	1	20	2715	2734	UGACAGAAGAGAGUGAGCAC	::::.:::::.:: :: :.:	UUGCUUACUCUUUUCUUUUA	Cleavage	reverse complement	1
miR156c	*ZmFAR1-5*	3	-1	1	20	2715	2734	UGACAGAAGAGAGUGAGCAC	::::.:::::.:: :: :.:	UUGCUUACUCUUUUCUUUUA	Cleavage	reverse complement	1
miR156d-5p	*ZmFAR1-5*	3	-1	1	20	2715	2734	UGACAGAAGAGAGUGAGCAC	::::.:::::.:: :: :.:	UUGCUUACUCUUUUCUUUUA	Cleavage	reverse complement	1
miR156e-5p	*ZmFAR1-5*	3	-1	1	20	2715	2734	UGACAGAAGAGAGUGAGCAC	::::.:::::.:: :: :.:	UUGCUUACUCUUUUCUUUUA	Cleavage	reverse complement	1
miR156f-5p	*ZmFAR1-5*	3	-1	1	20	2715	2734	UGACAGAAGAGAGUGAGCAC	::::.:::::.:: :: :.:	UUGCUUACUCUUUUCUUUUA	Cleavage	reverse complement	1
miR156g-5p	*ZmFAR1-5*	3	-1	1	20	2715	2734	UGACAGAAGAGAGUGAGCAC	::::.:::::.:: :: :.:	UUGCUUACUCUUUUCUUUUA	Cleavage	reverse complement	1
miR156h-5p	*ZmFAR1-5*	3	-1	1	20	2715	2734	UGACAGAAGAGAGUGAGCAC	::::.:::::.:: :: :.:	UUGCUUACUCUUUUCUUUUA	Cleavage	reverse complement	1
miR156i-5p	*ZmFAR1-5*	3	-1	1	20	2715	2734	UGACAGAAGAGAGUGAGCAC	::::.:::::.:: :: :.:	UUGCUUACUCUUUUCUUUUA	Cleavage	reverse complement	1
miR156l-5p	*ZmFAR1-5*	3	-1	1	20	2715	2734	UGACAGAAGAGAGUGAGCAC	::::.:::::.:: :: :.:	UUGCUUACUCUUUUCUUUUA	Cleavage	reverse complement	1
miR160a-3p	*ZmFAR1-2*	3	-1	1	21	2527	2547	GCGUGCAAGGGGCCAAGCAUG	: : :::::.: ::::.::	UCUUCAUGGCCCUUUGCAUGC	Cleavage	reverse complement	1
miR160a-5p	*ZmFAR1-2*	3	-1	1	21	6766	6786	UGCCUGGCUCCCUGUAUGCCA	::::.:: ::.: ::::::.	ACGCAUGCACGGGGCCAGGCG	Cleavage	reverse complement	1
miR160b-5p	*ZmFAR1-2*	3	-1	1	21	6766	6786	UGCCUGGCUCCCUGUAUGCCA	::::.:: ::.: ::::::.	ACGCAUGCACGGGGCCAGGCG	Cleavage	reverse complement	1
miR160c-5p	*ZmFAR1-2*	3	-1	1	21	6766	6786	UGCCUGGCUCCCUGUAUGCCA	::::.:: ::.: ::::::.	ACGCAUGCACGGGGCCAGGCG	Cleavage	reverse complement	1

miR160d-5p	*ZmFAR1-2*	3	-1	1	21	6766	6786	UGCCUGGCUCCCUGUAUGCCA	::::.:: ::.: ::::::.	ACGCAUGCACGGGGCCAGGCG	Cleavage	reverse complement	1
miR160e	*ZmFAR1-2*	3	-1	1	21	6766	6786	UGCCUGGCUCCCUGUAUGCCA	::::.:: ::.: ::::::.	ACGCAUGCACGGGGCCAGGCG	Cleavage	reverse complement	1
miR160f-5p	*ZmFAR1-2*	3	-1	1	21	6766	6786	UGCCUGGCUCCCUGUAUGCCG	::::.:: ::.: ::::::.	ACGCAUGCACGGGGCCAGGCG	Cleavage	reverse complement	1
miR160g-5p	*ZmFAR1-2*	3	-1	1	21	6766	6786	UGCCUGGCUCCCUGUAUGCCA	::::.:: ::.: ::::::.	ACGCAUGCACGGGGCCAGGCG	Cleavage	reverse complement	1
miR159e-3p	*ZmFAR1-9*	3.5	-1	1	21	4627	4647	AUUGGUUUGAAGGGAGCUCCA	:::::: .::: ::...:	CGGAGCUCAUUUCAAAUUGAU	Cleavage		1
miR168a-3p	*ZmFAR1-8*	3.5	-1	1	20	626	645	CCCGCCUUGCACCAAGUGAA	:.:. ::::: :::.::	GUUAUCAGGUGCAAGGUGGG	Cleavage	reverse complement	1
miR396a-3p	*ZmFAR1-11*	3.5	-1	1	21	2648	2668	GUUCAAUAAAGCUGUGGGAAA	::....:: ::::: :::::.	AUUUUUACCGCUUUAUUGAAU	Cleavage		1
miR396b-3p	*ZmFAR1-11*	3.5	-1	1	21	2648	2668	GUUCAAUAAAGCUGUGGGAAA	::....:: ::::: :::::.	AUUUUUACCGCUUUAUUGAAU	Cleavage		1
miR827-5p	*ZmFAR1-5*	3.5	-1	1	21	947	967	UUUGUUGGUGGUCAUUUAACC	: ::.:: :::: :.::::	GUUUGAACAACCACCGACAAA	Cleavage	reverse complement	1
miR156j-5p	*ZmFAR1-5*	4	-1	1	21	2714	2734	UGACAGAAGAGAGAGAGCACA	::::. ::::.: ::: :.:	AUUGCUUACUCUUUUCUUUUA	Cleavage	reverse complement	1
miR156k-5p	*ZmFAR1-5*	4	-1	1	20	2715	2734	UGACAGAAGAGAGCGAGCAC	::::. ::::.:: :: :.:	UUGCUUACUCUUUUCUUUUA	Cleavage	reverse complement	1
miR166b-5p	*ZmFAR1-15*	4	-1	1	21	1350	1370	GGAAUGUUGUCUGGUUCAAGG	:: ::.::: :::::::.	GGUUUUACUAGACAACAUUUG	Cleavage	reverse complement	1
miR166d-5p	*ZmFAR1-15*	4	-1	1	21	1350	1370	GGAAUGUUGUCUGGUUCAAGG	:: ::.::: :::::::.	GGUUUUACUAGACAACAUUUG	Cleavage	reverse complement	1
miR166g-5p	*ZmFAR1-15*	4	-1	1	21	1350	1370	GGAAUGUUGUCUGGUUGGAGA	:.. ::.:::: :::::.	GGUUUUACUAGACAACAUUUG	Cleavage	reverse complement	2
miR167a-5p	*ZmFAR1-12*	4	-1	1	21	3486	3506	UGAAGCUGCCAGCAUGAUCUA	: ..::.::: .::::::	ACUAAUGUGUUGGUAGCUUCA	Cleavage		1
miR167b-3p	*ZmFAR1-9*	4	-1	1	23	4051	4073	GAUCAUGCUGUGACAGUUUCACU	: ::::: :::::. :::	UUAUACACUGUCACAGCGAGAUG	Cleavage		1

miR167b-5p	*ZmFAR1-12*	4	-1	1	21	3486	3506	UGAAGCUGCCAGCAUGAUCUA	: ..::.:::<br>.::::::	ACUAAUGUGUUGGUAGCUUCA	Cleavage		1
miR167c-5p	*ZmFAR1-12*	4	-1	1	21	3486	3506	UGAAGCUGCCAGCAUGAUCUA	: ..::.:::<br>.::::::	ACUAAUGUGUUGGUAGCUUCA	Cleavage		1
miR167d-5p	*ZmFAR1-12*	4	-1	1	21	3486	3506	UGAAGCUGCCAGCAUGAUCUA	: ..::.:::<br>.::::::	ACUAAUGUGUUGGUAGCUUCA	Cleavage		1
miR167e-5p	*ZmFAR1-12*	4	-1	1	21	3486	3506	UGAAGCUGCCAGCAUGAUCUG	: ..::.:::<br>.::::::	ACUAAUGUGUUGGUAGCUUCA	Cleavage		1
miR167f-5p	*ZmFAR1-12*	4	-1	1	21	3486	3506	UGAAGCUGCCAGCAUGAUCUG	: ..::.:::<br>.::::::	ACUAAUGUGUUGGUAGCUUCA	Cleavage		1
miR167g-5p	*ZmFAR1-12*	4	-1	1	21	3486	3506	UGAAGCUGCCAGCAUGAUCUG	: ..::.:::<br>.::::::	ACUAAUGUGUUGGUAGCUUCA	Cleavage		1
miR167h-5p	*ZmFAR1-12*	4	-1	1	21	3486	3506	UGAAGCUGCCAGCAUGAUCUG	: ..::.:::<br>.::::::	ACUAAUGUGUUGGUAGCUUCA	Cleavage		1
miR167i-5p	*ZmFAR1-12*	4	-1	1	21	3486	3506	UGAAGCUGCCAGCAUGAUCUG	: ..::.:::<br>.::::::	ACUAAUGUGUUGGUAGCUUCA	Cleavage		1
miR167j-5p	*ZmFAR1-12*	4	-1	1	21	3486	3506	UGAAGCUGCCAGCAUGAUCUG	: ..::.:::<br>.::::::	ACUAAUGUGUUGGUAGCUUCA	Cleavage		1
miR168b-3p	*ZmFAR1-8*	4	-1	1	20	626	645	CCCGCCUUGCAUCAAGUGAA	:.:. :.:::<br>:::.:::	GUUAUCAGGUGCAAGGUGGG	Cleavage	reverse complement	1
miR2275c-5p	*ZmFAR1-4*	4	-1	1	21	5570	5590	AGGAUUAGAGGGACUUGAACC	:::: ::..<br>:::.:::.:	UUUUCACAUCUUUCUGAUCUU	Cleavage	reverse complement	1
miR395m-5p	*ZmFAR1-12*	4	-1	1	22	2547	2568	GUUCCUUUCAAACACUUCACAU	: : :: :::::<br>:.:::.:	CUUUAAAAUGUUUGAGAGGGAA	Cleavage		1
miR398a-5p	*ZmFAR1-4*	4	-1	1	21	6091	6111	GGGGCGAACUGAGAACACAUG	: : :::::::<br>:: :::.	UCUCUUUUCUCAGUUCUCCCU	Cleavage	reverse complement	1
miR399b-5p	*ZmFAR1-13*	4	-1	1	21	325	345	GUGCAGCUCUCCUCUGGCAUG	: :.::::.:<br>::::::	AGGGGCGGAGGGGAGCUGCAG	Cleavage		1
miR399f-3p	*ZmFAR1-8*	4	-1	1	21	849	869	UGCCAAAGGAAAUUUGCCCCG	: : :::::<br>.:::::.	GAUGACUAAUUUCUUUUGGCG	Cleavage	reverse complement	1
miR482-3p	*ZmFAR1-10*	4	-1	1	20	3851	3870	UCUUCCUUGUUCCUCCCAUU	: ::::::::.:::<br>:.::.	AUUGGGAGGAGCAAAGGAGG	Cleavage		2

miR156a-5p	*ZmFAR1-4*	4.5	-1	1	20	6083	6102	UGACAGAA<br>GAGAGUGA<br>GCAC	::. .:.:::::.<br>: :::	CUGUGUA<br>UUCUCUU<br>UUCUCA	Cleavage	reverse<br>complement	1
miR156b-5p	*ZmFAR1-4*	4.5	-1	1	20	6083	6102	UGACAGAA<br>GAGAGUGA<br>GCAC	::. .:.:::::.<br>: :::	CUGUGUA<br>UUCUCUU<br>UUCUCA	Cleavage	reverse<br>complement	1
miR156c	*ZmFAR1-4*	4.5	-1	1	20	6083	6102	UGACAGAA<br>GAGAGUGA<br>GCAC	::. .:.:::::.<br>: :::	CUGUGUA<br>UUCUCUU<br>UUCUCA	Cleavage	reverse<br>complement	1
miR156d-5p	*ZmFAR1-4*	4.5	-1	1	20	6083	6102	UGACAGAA<br>GAGAGUGA<br>GCAC	::. .:.:::::.<br>: :::	CUGUGUA<br>UUCUCUU<br>UUCUCA	Cleavage	reverse<br>complement	1
miR156e-5p	*ZmFAR1-4*	4.5	-1	1	20	6083	6102	UGACAGAA<br>GAGAGUGA<br>GCAC	::. .:.:::::.<br>: :::	CUGUGUA<br>UUCUCUU<br>UUCUCA	Cleavage	reverse<br>complement	1
miR156f-5p	*ZmFAR1-4*	4.5	-1	1	20	6083	6102	UGACAGAA<br>GAGAGUGA<br>GCAC	::. .:.:::::.<br>: :::	CUGUGUA<br>UUCUCUU<br>UUCUCA	Cleavage	reverse<br>complement	1
miR156g-5p	*ZmFAR1-4*	4.5	-1	1	20	6083	6102	UGACAGAA<br>GAGAGUGA<br>GCAC	::. .:.:::::.<br>: :::	CUGUGUA<br>UUCUCUU<br>UUCUCA	Cleavage	reverse<br>complement	1
miR156h-5p	*ZmFAR1-4*	4.5	-1	1	20	6083	6102	UGACAGAA<br>GAGAGUGA<br>GCAC	::. .:.:::::.<br>: :::	CUGUGUA<br>UUCUCUU<br>UUCUCA	Cleavage	reverse<br>complement	1
miR156i-3p	*ZmFAR1-4*	4.5	-1	1	22	6417	6438	GCUCACUG<br>CUCUAUCU<br>GUCAUC	: :.::::: ::.<br>: ::.::	CAGGGCA<br>GAUAUAG<br>UAAUGGG<br>C	Translatio<br>n	reverse<br>complement	1
miR156i-5p	*ZmFAR1-4*	4.5	-1	1	20	6083	6102	UGACAGAA<br>GAGAGUGA<br>GCAC	::. .:.:::::.<br>: :::	CUGUGUA<br>UUCUCUU<br>UUCUCA	Cleavage	reverse<br>complement	1
miR156j-5p	*ZmFAR1-15*	4.5	-1	1	21	4394	4414	UGACAGAA<br>GAGAGAGA<br>GCACA	:: :.:.:.:<br>:::: ::	CCUGGUU<br>UUUUUUU<br>UCUGGCA	Cleavage	reverse<br>complement	1
miR156l-3p	*ZmFAR1-4*	4.5	-1	1	22	6417	6438	GCUCACUG<br>CUCUAUCU<br>GUCACC	:.::::: :<br>:.: ::.::	CAGGGCA<br>GAUAUAG<br>UAAUGGG<br>C	Translatio<br>n	reverse<br>complement	1
miR156l-5p	*ZmFAR1-4*	4.5	-1	1	20	6083	6102	UGACAGAA<br>GAGAGUGA<br>GCAC	::. .:.:::::.<br>: :::	CUGUGUA<br>UUCUCUU<br>UUCUCA	Cleavage	reverse<br>complement	1
miR159a-3p	*ZmFAR1-10*	4.5	-1	1	21	7993	8013	UUUGGAUU<br>GAAGGGAG<br>CUCUG	: :::: :::.<br>::::::.::	AACAGCU<br>GGCUUUA<br>AUCCGAA	Cleavage		1
miR159b-3p	*ZmFAR1-10*	4.5	-1	1	21	7993	8013	UUUGGAUU<br>GAAGGGAG<br>CUCUG	: :::: :::.<br>::::::.::	AACAGCU<br>GGCUUUA<br>AUCCGAA	Cleavage		1
miR159e-3p	*ZmFAR1-16*	4.5	-1	1	21	700	720	AUUGGUUU<br>GAAGGGAG<br>CUCCA	: : : .::::<br>.:::::.:	GAGUGAU<br>GUCUUCA<br>GACCAGU	Cleavage		1

miR159e-5p	*ZmFAR1-15*	4.5	-1	1	21	318	338	CAGCUCCUGCAGCAUCUGUUC	:.: :::: : .:::::::	UCUCGGCUGCUCCGGGAGCUG	Translation	reverse complement	1
miR159f-3p	*ZmFAR1-10*	4.5	-1	1	21	7993	8013	UUUGGAUUGAAGGGAGCUCUG	: :::: :::. ::::::.::	AACAGCUGGCUUUAAUCCGAA	Cleavage		1
miR159h-3p	*ZmFAR1-10*	4.5	-1	1	21	2981	3001	UUUGGAGUGAAGGGAGCUCUG	.: ::: ..::.:: :::::..	GGGUGCUAUUUUUACUCCAGG	Cleavage		1
miR159i-3p	*ZmFAR1-10*	4.5	-1	1	21	2981	3001	UUUGGAGUGAAGGGAGCUCUG	.: ::: ..::.:: :::::..	GGGUGCUAUUUUUACUCCAGG	Cleavage		1
miR159j-3p	*ZmFAR1-10*	4.5	-1	1	21	7993	8013	UUUGGAUUGAAGGGAGCUCUG	: :::: :::. ::::::.::	AACAGCUGGCUUUAAUCCGAA	Cleavage		1
miR159k-3p	*ZmFAR1-10*	4.5	-1	1	21	7993	8013	UUUGGAUUGAAGGGAGCUCUG	: :::: :::. ::::::.::	AACAGCUGGCUUUAAUCCGAA	Cleavage		1
miR160b-3p	*ZmFAR1-2*	4.5	-1	1	21	2527	2547	GCGUGCAAGGAGCCAAGCAUG	: : :::: :.: :::::.::	UCUUCAUGGCCCUUUGCAUGC	Translation	reverse complement	1
miR160g-3p	*ZmFAR1-2*	4.5	-1	1	21	2527	2547	GCGUGCAAGGAGCCAAGCAUG	: : :::: :.: :::::.::	UCUUCAUGGCCCUUUGCAUGC	Translation	reverse complement	1
miR164g-3p	*ZmFAR1-10*	4.5	-1	1	21	6379	6399	CACGUGCUCCCCUUCUCCACC	:: ::.:::: .:: ::::	AAAGGCGAGGGGGGGCUCGUG	Cleavage		1
miR166a-5p	*ZmFAR1-5*	4.5	-1	1	21	4004	4024	GGAAUGUUGUCUGGCUCGGGG	.:::::::: ::.::..	AGGUGAGCCAGACCACGUUUU	Cleavage	reverse complement	1
miR166b-5p	*ZmFAR1-5*	4.5	-1	1	21	4004	4024	GGAAUGUUGUCUGGUUCAAGG	:::.:::::: ::.::..	AGGUGAGCCAGACCACGUUUU	Cleavage	reverse complement	1
miR166c-5p	*ZmFAR1-5*	4.5	-1	1	21	4004	4024	GGAAUGUUGUCUGGCUCGAGG	.:::::::: ::.::..	AGGUGAGCCAGACCACGUUUU	Cleavage	reverse complement	1
miR166d-5p	*ZmFAR1-5*	4.5	-1	1	21	4004	4024	GGAAUGUUGUCUGGUUCAAGG	:::.:::::: ::.::..	AGGUGAGCCAGACCACGUUUU	Cleavage	reverse complement	1
miR166g-5p	*ZmFAR1-15*	4.5	-1	1	21	3746	3766	GGAAUGUUGUCUGGUUGGAGA	:::..::::. ::: :::.	GAGCCAGUCAGAUAACCUUCU	Cleavage	reverse complement	2
miR166j-5p	*ZmFAR1-12*	4.5	-1	1	22	1541	1562	GGUUUGUUUGUCUGGUUCAAGG	:: .:.:::.:: .:.:.:.:	GAUUUGAUCAGGCAGAUAGAUC	Cleavage		1
miR166j-5p	*ZmFAR1-3*	4.5	-1	1	22	2656	2677	GGUUUGUUUGUCUGGUUCAAGG	: :: :.:::...: :..:::.	GCAUGUAUCAGGUGAAUGAACU	Cleavage		1

miR166m-5p	*ZmFAR1-4*	4.5	-1	1	21	2267	2287	GGAAUGUUGGCUGGCUCGAGG	: :: : :::: :: :.:::.	CAUCAACCCAGCAAACGUUCU	Cleavage	reverse complement	1
miR167e-3p	*ZmFAR1-5*	4.5	-1	1	22	3341	3362	GAUCAUGCUGUGCAGUUUCAUC	::::: :.:: .:.::::	AAUGAAAGAGUACGGUAUGAUG	Cleavage	reverse complement	1
miR168a-5p	*ZmFAR1-3*	4.5	-1	1	21	265	285	UCGCUUGGUGCAGAUCGGGAC	::::. .:::::: : :::.:.	GUCCUCGUCUGCAACAAGUGG	Cleavage		1
miR168b-5p	*ZmFAR1-3*	4.5	-1	1	21	265	285	UCGCUUGGUGCAGAUCGGGAC	::::. .:::::: : :::.:.	GUCCUCGUCUGCAACAAGUGG	Cleavage		1
miR169f-3p	*ZmFAR1-4*	4.5	-1	1	21	5883	5903	GGCAUGUCUUCCUUGGCUACU	:::: ..:::::: ::. ::.	CGUAGAUGAGGAAGACGAGCU	Cleavage	reverse complement	1
miR169n-3p	*ZmFAR1-16*	4.5	-1	1	20	1893	1912	GGCAGGCCUUCUUGGCUAAG	.::.: ::.::: :.: ::.:	UUUGGGCAGGAAUGUCUGUC	Cleavage		1
miR169q-3p	*ZmFAR1-4*	4.5	-1	1	19	2471	2489	GGCAGGCCUUCUGGCUAAG	.::.:...:. :::: :::	UUUGGUUGGGUGGCCUGCC	Cleavage	reverse complement	1
miR171g-5p	*ZmFAR1-11*	4.5	-1	1	21	3035	3055	UAUUGACUUGGCUCAUCUCUC	: :. ::: :::::: :...::	GUGGAAUGUGCCAAGUUGGUA	Cleavage		1
miR171n-5p	*ZmFAR1-7*	4.5	-1	1	21	1864	1884	UAUUGGUGAGGUUCAAUCCGA	::: .: :.:::: ::::.::	UUGAGUACAGCCUCACCAGUA	Cleavage	reverse complement	1
miR2275b-5p	*ZmFAR1-10*	4.5	-1	1	21	7958	7978	AGGAUUAGAGGCAACUGAACC	: :::. ::: ::.: ::::	GCUUCGCUUGGCUUUAAUCCG	Translation		1
miR2275c-5p	*ZmFAR1-15*	4.5	-1	1	21	4375	4395	AGGAUUAGAGGGACUUGAACC	:::::.:::::. .: .::::	GGUUCGAGUCCUGUUGAUCCC	Cleavage	reverse complement	1
miR2275c-5p	*ZmFAR1-5*	4.5	-1	1	21	3231	3251	AGGAUUAGAGGGACUUGAACC	: :::::::.:. :::::	AUUCCAAGUCCUUUUAAUCAG	Cleavage	reverse complement	1
miR2275c-5p	*ZmFAR1-7*	4.5	-1	1	21	596	616	AGGAUUAGAGGGACUUGAACC	:::.::::::::. :.::.	AAUUCGAGUCCCUUGAGUCUG	Cleavage	reverse complement	1
miR2275c-5p	*ZmFAR1-9*	4.5	-1	1	21	5119	5139	AGGAUUAGAGGGACUUGAACC	::::: :::.:: :.:::	UAUUCAACUCCUUCAAGUCCC	Cleavage		1
miR2275d-3p	*ZmFAR1-9*	4.5	-1	1	22	4569	4590	UUUGUUUUCCUCUAAUAUCUCA	::::.:::::: :.::: :.:	ACAGAUGUUAGAGAAGAAGAGA	Cleavage		1
miR2275d-3p	*ZmFAR1-2*	4.5	-1	1	22	4415	4436	UUUGUUUUCCUCUAAUAUCUCA	: ::: :: : :::::::	UCUGCUAUGAGUGG	Translation	reverse complement	1

									AAAACAA<br>C			
miR2275d-5p	*ZmFAR1-2*	4.5	-1	1	21	1832	1852	AGAGUUGG<br>AGGAAAGA<br>AACU	: ::::::::<br>:. ::::	UUUCUUC<br>UUUCCUC<br>UCCCUCU	Cleavage	reverse complement	1
miR394a-3p	*ZmFAR1-6*	4.5	-1	1	20	2845	2864	AGGUGGGC<br>AUACUGCC<br>AAUG	::: ::: :::<br>.::.:::	AAUUUUC<br>AGAAUGU<br>CCGCCU	Translation		1
miR394b-3p	*ZmFAR1-6*	4.5	-1	1	20	2845	2864	AGGUGGGC<br>AUACUGCC<br>AAUG	::: ::: :::<br>.::.:::	AAUUUUC<br>AGAAUGU<br>CCGCCU	Translation		1
miR395a-5p	*ZmFAR1-3*	4.5	-1	1	23	3105	3127	GUUCUCCU<br>CAAACCAC<br>UUCAGUU	::.::::<br>: ::::.:::.	UUAGGAG<br>GUGGAUC<br>GAGGGGA<br>AU	Translation		1
miR395c-3p	*ZmFAR1-4*	4.5	-1	1	21	1922	1942	GUGAAGUG<br>UUUGGAGG<br>AACUC	::.::::.<br>:::.::::	CAACUCU<br>UCCAGAC<br>AUUUCAG	Cleavage	reverse complement	1
miR395k-3p	*ZmFAR1-4*	4.5	-1	1	21	3103	3123	GUGAAGUG<br>UUUGAGGA<br>AACUC	::::::.::.<br>...::::	AUAUUUC<br>CUUAAGU<br>GUUUCAA	Cleavage	reverse complement	1
miR395l-3p	*ZmFAR1-4*	4.5	-1	1	21	1922	1942	GUGAAGUG<br>UUUGGAGG<br>AACUC	::.::::.<br>:::.::::	CAACUCU<br>UCCAGAC<br>AUUUCAG	Cleavage	reverse complement	1
miR395m-3p	*ZmFAR1-4*	4.5	-1	1	21	1922	1942	GUGAAGUG<br>UUUGGAGG<br>AACUC	::.::::.<br>:::.::::	CAACUCU<br>UCCAGAC<br>AUUUCAG	Cleavage	reverse complement	1
miR395o-3p	*ZmFAR1-14*	4.5	-1	1	21	346	366	GUGAAGUG<br>UUUGGGUG<br>AACUC	:: :::::: :.<br>::::::	AUGUACA<br>CCCAUAU<br>CCUUCAC	Translation		1
miR396c	*ZmFAR1-1*	4.5	-1	1	22	4301	4322	UUCCACAG<br>GCUUUCUU<br>GAACUG	.: : ::::: .:::<br>::::::	UAAUACA<br>AGAUGGC<br>CAGUGGA<br>A	Cleavage	reverse complement	1
miR396d	*ZmFAR1-1*	4.5	-1	1	22	4301	4322	UUCCACAG<br>GCUUUCUU<br>GAACUG	.: : ::::: .:::<br>::::::	UAAUACA<br>AGAUGGC<br>CAGUGGA<br>A	Cleavage	reverse complement	1
miR396e-3p	*ZmFAR1-6*	4.5	-1	1	21	814	834	GGUCAAGA<br>AAGCCGUG<br>GGAAG	:::: ::: :<br>:::::::..	ACGCCCA<br>GGGCCUU<br>CUUGAUU	Translation		1
miR396g-3p	*ZmFAR1-11*	4.5	-1	1	21	2648	2668	GUUCAAGA<br>AAGCUGUG<br>GAAGA	.::..:: ::::<br>:::::.	AUUUUUA<br>CCGCUUU<br>AUUGAAU	Cleavage		1
miR397a-3p	*ZmFAR1-10*	4.5	-1	1	22	256	277	UAGCCGUU<br>AGCGCUCA<br>UUAACU	::.:: :.:<br>::::: ::::	GUUUGAU<br>AUGUGCU<br>AACAGCU<br>A	Cleavage		1
miR399b-5p	*ZmFAR1-10*	4.5	-1	1	21	8952	8972	GUGCAGCU<br>CUCCUCUG<br>GCAUG	::: ::: :::::::<br>:: .:.	CAUACCA<br>AAGGAGA<br>GCUCUAU	Cleavage		1

miR399e-5p	*ZmFAR1-4*	4.5	-1	1	21	5625	5645	GGGCUUCU CUUUCUUG GCAGG	.:::::: :.:::. ::::..	UCUGCCA UGGAAGG CAAGCUU	Cleavage	reverse complement	1
miR399h-5p	*ZmFAR1-13*	4.5	-1	1	21	325	345	GUGCAGUU CUCCUCUG GCACG	: : :.::::.::. :::::	AGGGGCG GAGGGGA GCUGCAG	Cleavage		1
miR399i-5p	*ZmFAR1-13*	4.5	-1	1	21	325	345	GUGCGGCU CUCCUCUG GCAUG	: :.::::.: :::.:::	AGGGGCG GAGGGGA GCUGCAG	Cleavage		1
miR528a-5p	*ZmFAR1-4*	4.5	-1	1	21	966	986	UGGAAGGG GCAUGCAG AGGAG	::::: :: ::: .::::.	ACCCUCU CCAGGCC UCUUCUC	Translatio n	reverse complement	1
miR528b-5p	*ZmFAR1-4*	4.5	-1	1	21	966	986	UGGAAGGG GCAUGCAG AGGAG	::::: :: ::: .::::.	ACCCUCU CCAGGCC UCUUCUC	Translatio n	reverse complement	1
miR529-3p	*ZmFAR1-16*	4.5	-1	1	21	629	649	GCUGUACC CUCUCUCU UCUUC	.::::::. :: :::.:::	AUGGAAG AGGAAGA GUAUAGC	Translatio n		1
miR529-5p	*ZmFAR1-4*	4.5	-1	1	21	6080	6100	AGAAGAGA GAGAGUAC AGCCU	:: ::::.. .:::: :.::::	AGCCUGU GUAUUCU CUUUUCU	Cleavage	reverse complement	1
miR827-5p	*ZmFAR1-12*	4.5	-1	1	21	371	391	UUUGUUGG UGGUCAUU UAACC	: : :::: ::.::: :: :::	GUUCAAA UUACUAC CAAAAAA	Cleavage		1
miR1432-5p	*ZmFAR1-6*	5	-1	1	21	2616	2636	CUCAGGAG AGAUGACA CCGAC	::. ::::::: ::.. :::	GUUUUUG UCAUCUC UUUGGAG	Cleavage		1
miR1432-5p	*ZmFAR1-13*	5	-1	1	21	3976	3996	CUCAGGAG AGAUGACA CCGAC	: :: :::::: :: :::	AACAGUC UCAUCUC UCACGAG	Cleavage		1
miR156d-3p	*ZmFAR1-12*	5	-1	1	22	944	965	GCUCACUU CUCUUUCU GUCAGC	:. ::::. : :::: ::::	UUCGGGA GAAGAAG AAGGGAG C	Translatio n		1
miR156f-3p	*ZmFAR1-12*	5	-1	1	22	944	965	GCUCACUU CUCUUUCU GUCAGC	:. ::::. : :::: ::::	UUCGGGA GAAGAAG AAGGGAG C	Translatio n		1
miR156g-3p	*ZmFAR1-12*	5	-1	1	22	944	965	GCUCACUU CUCUUUCU GUCAGC	:. ::::. : :::: ::::	UUCGGGA GAAGAAG AAGGGAG C	Translatio n		1
miR156i-3p	*ZmFAR1-3*	5	-1	1	22	2939	2960	GCUCACUG CUCUAUCU GUCAUC	::::::: :: :::::	UUCUACA GAUAGAG GAGUGAC U	Cleavage		1
miR156j-5p	*ZmFAR1-4*	5	-1	1	21	627	647	UGACAGAA GAGAGAGA GCACA	: : ::.:::.: ::: ::.	AUUCCCC UUUCUUU UCUUUCG	Cleavage	reverse complement	1

miR156k-3p	*ZmFAR1-10*	5	-1	1	22	5376	5397	GCUCGCUUCUCUUUCUGUCAGC	:.:.: .::.<br>::.:::.:	GGCGGCGGCGAGGGAGGCGGGG	Cleavage		1
miR156k-3p	*ZmFAR1-12*	5	-1	1	22	944	965	GCUCGCUUCUCUUUCUGUCAGC	:. ::::. :<br>:::: ::::	UUCGGGAGAAGAAGAAGGGAGC	Translation		1
miR156l-3p	*ZmFAR1-3*	5	-1	1	22	2939	2960	GCUCACUGCUCUAUCUGUCACC	:::::::<br>:: :::::	UUCUACAGAUAGAGGAGUGACU	Cleavage		1
miR159b-5p	*ZmFAR1-15*	5	-1	1	21	480	500	GUGCUCCCUUCAAACCAAUAA	: ::::: ::: ::<br>:.::.:	UGAUUGGAUUGCAGAGGGCGC	Translation	reverse complement	1
miR159e-3p	*ZmFAR1-5*	5	-1	1	21	4855	4875	AUUGGUUUGAAGGGAGCUCCA	:.:: .::::..<br>.:.:::	CCGGGCCUCCUUUGGAUCAAA	Cleavage	reverse complement	1
miR159g-3p	*ZmFAR1-5*	5	-1	1	21	582	602	UUUGGAGUGAAGGGAGUUCUG	::.::::..: ...<br>:::.::	GAGGACUCUUUCUGUUCCGAA	Translation	reverse complement	2
miR159g-3p	*ZmFAR1-5*	5	-1	1	21	4045	4065	UUUGGAGUGAAGGGAGUUCUG	:.:::: ::.:: ::<br>::.::	CGGAACGCCUUUGACCCCGAA	Cleavage	reverse complement	2
miR159g-3p	*ZmFAR1-8*	5	-1	1	21	367	387	UUUGGAGUGAAGGGAGUUCUG	::.::: :.: ..:<br>:::::.	AAGGACUACUUCUGCUCCAAG	Translation	reverse complement	1
miR159g-3p	*ZmFAR1-2*	5	-1	1	21	3301	3321	UUUGGAGUGAAGGGAGUUCUG	.: :::: : :..<br>::::::	AGGUACUCCAUCCGUUCCAAA	Translation	reverse complement	1
miR159g-3p	*ZmFAR1-10*	5	-1	1	21	2981	3001	UUUGGAGUGAAGGGAGUUCUG	.: .:: ..::.::<br>::::..	GGGUGCUAUUUUUACUCCAGG	Cleavage		1
miR159h-3p	*ZmFAR1-6*	5	-1	1	21	507	527	UUUGGAGUGAAGGGAGCUCUG	:::: :.: ::..:<br>::::..	AAGAGGUUCGUUUGCUCCAGG	Cleavage		1
miR159i-3p	*ZmFAR1-6*	5	-1	1	21	507	527	UUUGGAGUGAAGGGAGCUCUG	:::: :.: ::..:<br>::::..	AAGAGGUUCGUUUGCUCCAGG	Cleavage		1
miR159j-5p	*ZmFAR1-15*	5	-1	1	21	480	500	GUGCUCCCUUCAAACCAAUAA	: ::::: ::: ::<br>:.::.:	UGAUUGGAUUGCAGAGGGCGC	Translation	reverse complement	1
miR159k-5p	*ZmFAR1-15*	5	-1	1	21	480	500	GUGCUCCCUUCAAACCAAUAA	: ::::: ::: ::<br>:.::.:	UGAUUGGAUUGCAGAGGGCGC	Translation	reverse complement	1
miR160c-3p	*ZmFAR1-10*	5	-1	1	21	2794	2814	GCGUGCAUGGUGCCAAGCAUA	::.:: ::: .:<br>:::: ::	GUUGUUUUGCAAUAUGCAGGC	Translation		1

miR160c-3p	*ZmFAR1-9*	5	-1	1	21	6	26	GCGUGCAU GGUGCCAA GCAUA	.: :: :::: .: :::::	CGUCCUC CGCACCG UCCACGC	Cleavage		1
miR160f-3p	*ZmFAR1-2*	5	-1	1	21	2527	2547	GCGUGCGA GGUGCCAG GCAUG	: : :::: :.: .:::.::	UCUUCAU GGCCCUU UGCAUGC	Translation	reverse complement	1
miR164a-3p	*ZmFAR1-9*	5	-1	1	21	934	954	CACGUGUU CUCCUUCU CCAUC	:::::.:::: :.:: ::	CUGGGAG AGGGAGA GCAAAUG	Cleavage		1
miR164c-3p	*ZmFAR1-9*	5	-1	1	21	934	954	CAUGUGCC CUUCUUCU CCAUC	:::::.:.:: ::: :::	CUGGGAG AGGGAGA GCAAAUG	Cleavage		1
miR164d-3p	*ZmFAR1-9*	5	-1	1	20	1219	1238	CACGUGGU CUCCUUCU CCAU	:::.:::::::: :.:	UUGGGGA AGGAGAC GAUGAA	Cleavage		1
miR164d-3p	*ZmFAR1-2*	5	-1	1	20	1880	1899	CACGUGGU CUCCUUCU CCAU	:::: .:.::. :: :::	GGGGAGU GGGGGAU CAGGUG	Cleavage	reverse complement	1
miR164e-3p	*ZmFAR1-13*	5	-1	1	21	4230	4250	CAUGUGUC CGCCCUCU CCACC	:: :.: ::::: :: ..::	AGUUGGG UGGGCGG ACCUGUG	Cleavage		1
miR164h-3p	*ZmFAR1-9*	5	-1	1	21	934	954	CAUGUGCC CUUCUUCU CCAUC	:::::.:.:: ::: :::	CUGGGAG AGGGAGA GCAAAUG	Cleavage		1
miR166a-5p	*ZmFAR1-15*	5	-1	1	21	3746	3766	GGAAUGUU GUCUGGCU CGGGG	: ::.::::. ::: :::.	GAGCCAG UCAGAUA ACCUUCU	Cleavage	reverse complement	1
miR166c-5p	*ZmFAR1-15*	5	-1	1	21	3746	3766	GGAAUGUU GUCUGGCU CGAGG	: ::.::::. ::: :::.	GAGCCAG UCAGAUA ACCUUCU	Cleavage	reverse complement	1
miR166g-5p	*ZmFAR1-4*	5	-1	1	21	2116	2136	GGAAUGUU GUCUGGUU GGAGA	:::::: .:::: : ::: :.	UCUCCAA GUAGACC ACAUCCU	Cleavage	reverse complement	1
miR166g-5p	*ZmFAR1-5*	5	-1	1	21	2375	2395	GGAAUGUU GUCUGGUU GGAGA	:::.: :::::..:: .. :::	UCUUCUA CCAGGUA AUGAUCC	Cleavage	reverse complement	1
miR166g-5p	*ZmFAR1-10*	5	-1	1	21	3068	3088	GGAAUGUU GUCUGGUU GGAGA	:::.:: : ::.::::.:	UAGGCAA UCAAACA GCAUUUC	Translation		1
miR167b-3p	*ZmFAR1-1*	5	-1	1	23	3222	3244	GAUCAUGC UGUGACAG UUUCACU	: :.:::: ::: ..::.:.	UCCAACA UUGUCAC AUUGUGG UU	Cleavage	reverse complement	1
miR168a-5p	*ZmFAR1-8*	5	-1	1	21	337	357	UCGCUUGG UGCAGAUC GGGAC	:.. : : :::: ::::::.	UACUUCA CCGGCAC CAAGCGG	Cleavage	reverse complement	1
miR168b-5p	*ZmFAR1-8*	5	-1	1	21	337	357	UCGCUUGG UGCAGAUC GGGAC	:.. : : :::: ::::::.	UACUUCA CCGGCAC CAAGCGG	Cleavage	reverse complement	1

miR169a-5p	*ZmFAR1-7*	5	-1	1	21	1275	1295	CAGCCAAGGAUGACUUGCCGA	: :: ::..:. .:::::::	GCAGCCUGUUGUUUUUGGCUG	Cleavage	reverse complement	1
miR169b-5p	*ZmFAR1-7*	5	-1	1	21	1275	1295	CAGCCAAGGAUGACUUGCCGA	: :: ::..:. .:::::::	GCAGCCUGUUGUUUUUGGCUG	Cleavage	reverse complement	1
miR169c-5p	*ZmFAR1-7*	5	-1	1	21	1275	1295	CAGCCAAGGAUGACUUGCCGG	: :: ::..:. .:::::::	GCAGCCUGUUGUUUUUGGCUG	Cleavage	reverse complement	1
miR169f-3p	*ZmFAR1-6*	5	-1	1	21	4072	4092	GGCAUGUCUUCCUUGGCUACU	: ..:::::: ::: ::.:	UUGACUUAAGGAAGACUUGUC	Cleavage		1
miR169m-5p	*ZmFAR1-10*	5	-1	1	21	1480	1500	UAGCCAAGAAUGGCUUGCCUA	: :: :.::: : ::::::	GCAGAAACCUAUUCAUGGCUA	Cleavage		1
miR169n-5p	*ZmFAR1-10*	5	-1	1	21	1480	1500	UAGCCAAGAAUGGCUUGCCUA	: :: :.::: : ::::::	GCAGAAACCUAUUCAUGGCUA	Cleavage		1
miR169o-5p	*ZmFAR1-7*	5	-1	1	21	1275	1295	UAGCCAAGAAUGACUUGCCUA	:: ::.. ::.:::::.	GCAGCCUGUUGUUUUUGGCUG	Cleavage	reverse complement	1
miR169p-3p	*ZmFAR1-4*	5	-1	1	21	2040	2060	GGCAAGUCAUCUGGGGCUACG	: ::..::.:: :.:: .:	AAUUGCUUCAGGUGAUUUAUC	Cleavage	reverse complement	1
miR169q-5p	*ZmFAR1-10*	5	-1	1	21	1480	1500	UAGCCAAGAAUGGCUUGCCUA	: :: :.::: : ::::::	GCAGAAACCUAUUCAUGGCUA	Cleavage		1
miR169r-3p	*ZmFAR1-11*	5	-1	1	21	649	669	GGCAAGUUGUCCUUGGCUACA	:::::: . :: ::. ::::::	UGUAGCAGUGGCCAGCUUGCC	Translation		1
miR169r-5p	*ZmFAR1-7*	5	-1	1	21	1275	1295	CAGCCAAGGAUGACUUGCCGG	: :: ::..:. .:::::::	GCAGCCUGUUGUUUUUGGCUG	Cleavage	reverse complement	1
miR171a-5p	*ZmFAR1-1*	5	-1	1	21	3889	3909	UAUUGGCGAGGUUCAAUCAGA	.:::: : ::: .: .:::::	CUUGAUAGCACCAUGUCAAUA	Cleavage	reverse complement	1
miR171c-5p	*ZmFAR1-4*	5	-1	1	21	2023	2043	UAUUGGUGCGGUUCAAUCAGA	.::: :::..::.: ::::	CUUGAAUGAGUCGUAACAAUU	Cleavage	reverse complement	1
miR171f-5p	*ZmFAR1-9*	5	-1	1	21	3481	3501	CGAUGUUGGCAUGGCUCAAUC	: ::: .:::: :: :.::.:	AAAUGACUCAUGGCAAUAUUG	Cleavage		1
miR171h-5p	*ZmFAR1-14*	5	-1	1	21	2138	2158	UGGUAUUGUUUCGGCUCAUGU	:: .:..:::.: ::::.: :	GAAUUGGUUGAAGCAAUGCAA	Cleavage		1
miR171i-5p	*ZmFAR1-1*	5	-1	1	21	3889	3909	UGUUGGCACGGUUCAAUCAAA	:::: : ::: : :.:::.:	CUUGAUAGCACCAUGUCAAUA	Cleavage	reverse complement	1

miR171k-5p	*ZmFAR1-14*	5	-1	1	21	2138	2158	UGGUAUUG UUUCGGCU CAUGU	:: .:..:::.: ::::.: :	GAAUUGG UUGAAGC AAUGCAA	Cleavage		1
miR172a	*ZmFAR1-1*	5	-1	1	20	3341	3360	AGAAUCUU GAUGAUGC UGCA	.:: :: ::: :::::::	CCAGGCC UCUUCAA GAUUCU	Translatio n	reverse complement	1
miR172a	*ZmFAR1-10*	5	-1	1	20	4845	4864	AGAAUCUU GAUGAUGC UGCA	:: : :::::: .::::	AACAUCU UCAUCAA UGUUCU	Cleavage		1
miR172b-3p	*ZmFAR1-1*	5	-1	1	20	3341	3360	AGAAUCUU GAUGAUGC UGCA	.:: :: ::: :::::::	CCAGGCC UCUUCAA GAUUCU	Translatio n	reverse complement	1
miR172b-3p	*ZmFAR1-10*	5	-1	1	20	4845	4864	AGAAUCUU GAUGAUGC UGCA	:: : :::::: .::::	AACAUCU UCAUCAA UGUUCU	Cleavage		1
miR172c-3p	*ZmFAR1-1*	5	-1	1	20	3341	3360	AGAAUCUU GAUGAUGC UGCA	.:: :: ::: :::::::	CCAGGCC UCUUCAA GAUUCU	Translatio n	reverse complement	1
miR172c-3p	*ZmFAR1-10*	5	-1	1	20	4845	4864	AGAAUCUU GAUGAUGC UGCA	:: : :::::: .::::	AACAUCU UCAUCAA UGUUCU	Cleavage		1
miR172c-5p	*ZmFAR1-2*	5	-1	1	20	897	916	CAGCACCA CCAAGAUU CACA	::: .. .: ::::: ::::	UGUUGGG UUGGGUG GUGCUG	Translatio n	reverse complement	1
miR172d-3p	*ZmFAR1-1*	5	-1	1	20	3341	3360	AGAAUCUU GAUGAUGC UGCA	.:: :: ::: :::::::	CCAGGCC UCUUCAA GAUUCU	Translatio n	reverse complement	1
miR172d-3p	*ZmFAR1-10*	5	-1	1	20	4845	4864	AGAAUCUU GAUGAUGC UGCA	:: : :::::: .::::	AACAUCU UCAUCAA UGUUCU	Cleavage		1
miR2118a	*ZmFAR1-12*	5	-1	1	22	930	951	UUCCUGAU GCCUCUCA UUCCUA	::. :::.:: . ::.:::.	CUCGAGA GAGGGGU UUCGGGA G	Cleavage		1
miR2118f	*ZmFAR1-15*	5	-1	1	22	5207	5228	UUCCCAAU GCCUUCCA UGCCUA	:::::: ::::::: ::	GGUAGAU GGAAGGC AUUGUAA A	Cleavage	reverse complement	1
miR2118g	*ZmFAR1-15*	5	-1	1	22	3218	3239	UUCCUGAU GCCUCCUA UUCCUA	::::::::: :: : ::.	AGAGAAU AGGAGGC AAGAAGA G	Cleavage	reverse complement	1
miR2275a-3p	*ZmFAR1-10*	5	-1	1	22	930	951	UUUGUUUU CCUCCAAU AUCUCA	: :: .::: .::. :.:.:::	AGUGAAG UUGUGGG GAGAUAA A	Cleavage		1
miR2275a-3p	*ZmFAR1-5*	5	-1	1	22	4491	4512	UUUGUUUU CCUCCAAU AUCUCA	::.::: : ::::: .:::	CAUCAUG UUGUAGG AAACUAA A	Cleavage	reverse complement	1

miR2275a-5p	*ZmFAR1-13*	5	-1	1	21	1563	1583	AGAGUUGGAGGAAAGCAAACC	: : :.:::::: : :.:.:	UGGUCGUUUUCCUCCCAUUUU	Cleavage		1
miR2275a-5p	*ZmFAR1-2*	5	-1	1	21	2537	2557	AGAGUUGGAGGAAAGCAAACC	:::: : :.:. .::.:.:	CCUUUGCAUGCUUUUAAUUUU	Cleavage	reverse complement	1
miR2275a-5p	*ZmFAR1-4*	5	-1	1	21	632	652	AGAGUUGGAGGAAAGCAAACC	::: .::::.:. :. .:::	CCUUUCUUUUCUUUCGCUUCU	Cleavage	reverse complement	1
miR2275a-5p	*ZmFAR1-1*	5	-1	1	21	1000	1020	AGAGUUGGAGGAAAGCAAACC	:::: :.: ::::.: ::	AUUUUGCACUUCUCCAGCCCU	Cleavage	reverse complement	1
miR2275b-3p	*ZmFAR1-5*	5	-1	1	22	4491	4512	UUCAGUUUCCUCUAAUAUCUCA	::.::. : :::::: ::	CAUCAUGUUGUAGGAAACUAAA	Cleavage	reverse complement	1
miR2275b-3p	*ZmFAR1-15*	5	-1	1	22	3608	3629	UUCAGUUUCCUCUAAUAUCUCA	:.:. :.:: :::::::	GUUGGUGAUGGAUGAAACUGAU	Translation	reverse complement	1
miR2275b-5p	*ZmFAR1-1*	5	-1	1	21	1591	1611	AGGAUUAGAGGCAACUGAACC	::::::::: :: :.: :.	AGUUCAGUUGCAUCUGAGCUC	Translation	reverse complement	1
miR2275b-5p	*ZmFAR1-2*	5	-1	1	21	2537	2557	AGGAUUAGAGGCAACUGAACC	::.. :::. :.::::..:	CCUUUGCAUGCUUUUAAUUUU	Cleavage	reverse complement	1
miR2275b-5p	*ZmFAR1-12*	5	-1	1	21	1480	1500	AGGAUUAGAGGCAACUGAACC	: : ::: ::.:: :.:::.:	GCUGCAGCAGCUUCUGAUCUU	Cleavage		1
miR2275c-3p	*ZmFAR1-5*	5	-1	1	22	4491	4512	UUCAGUUUCCUCUAAUAUCUCA	::.::. : :::::: ::	CAUCAUGUUGUAGGAAACUAAA	Cleavage	reverse complement	1
miR2275c-3p	*ZmFAR1-15*	5	-1	1	22	3608	3629	UUCAGUUUCCUCUAAUAUCUCA	:.:. :.:: :::::::	GUUGGUGAUGGAUGAAACUGAU	Translation	reverse complement	1
miR2275d-5p	*ZmFAR1-4*	5	-1	1	21	632	652	AGAGUUGGAGGAAAGAAAACU	::: .::::.:. :. .:::	CCUUUCUUUUCUUUCGCUUCU	Cleavage	reverse complement	1
miR319a-3p	*ZmFAR1-16*	5	-1	1	20	1346	1365	UUGGACUGAAGGGUGCUCCC	::: ...:.::..: ::..:	AGGAAUGUCUUUUGGUCUGA	Cleavage		1
miR319a-5p	*ZmFAR1-15*	5	-1	1	20	481	500	GAGCUCUCUUCAGUCCACUC	:: ::::.:: :::: .:: :	GAUUGGAUUGCAGAGGGCGC	Translation	reverse complement	1
miR319b-3p	*ZmFAR1-16*	5	-1	1	20	1346	1365	UUGGACUGAAGGGUGCUCCC	::: ...:.::..: ::..:	AGGAAUGUCUUUUGGUCUGA	Cleavage		1

miR319c-3p	*ZmFAR1-16*	5	-1	1	20	1346	1365	UUGGACUGAAGGGUGCUCCC	::: ...:.::..: ::..:	AGGAAUGUCUUUUGGUCUGA	Cleavage		1
miR319c-5p	*ZmFAR1-15*	5	-1	1	20	481	500	GAGCUCUCUUCAGUCCACUC	:: ::::.:: :::: .:: :	GAUUGGAUUGCAGAGGGCGC	Translation	reverse complement	1
miR319d-3p	*ZmFAR1-16*	5	-1	1	20	1346	1365	UUGGACUGAAGGGUGCUCCC	::: ...:.::..: ::..:	AGGAAUGUCUUUUGGUCUGA	Cleavage		1
miR390a-5p	*ZmFAR1-4*	5	-1	1	21	1007	1027	AAGCUCAGGAGGGAUAGCGCC	:::: :..::: .: :::::	UGCGCACUUUCUCUUUAGCUU	Cleavage	reverse complement	1
miR390b-5p	*ZmFAR1-4*	5	-1	1	21	1007	1027	AAGCUCAGGAGGGAUAGCGCC	:::: :..::: .: :::::	UGCGCACUUUCUCUUUAGCUU	Cleavage	reverse complement	1
miR393a-3p	*ZmFAR1-7*	5	-1	1	22	1530	1551	AUCAGUGCAAUCCCUUUGGAAU	.: :::: : ::::.::::	UGAUCUAAGGAAGUGCAUUGAU	Translation	reverse complement	1
miR393a-5p	*ZmFAR1-6*	5	-1	1	22	2614	2635	UCCAAAGGGAUCGCAUUGAUCU	: .:. ::. ::: .::::::	ACGUUUUUGUCAUCUCUUUGGA	Cleavage		1
miR393b-5p	*ZmFAR1-6*	5	-1	1	22	2614	2635	UCCAAAGGGAUCGCAUUGAUCC	.:. ::. : ::.:::::::	ACGUUUUUGUCAUCUCUUUGGA	Cleavage		1
miR393c-5p	*ZmFAR1-6*	5	-1	1	22	2614	2635	UCCAAAGGGAUCGCAUUGAUCU	: .:. ::. ::: .::::::	ACGUUUUUGUCAUCUCUUUGGA	Cleavage		1
miR395a-3p	*ZmFAR1-4*	5	-1	1	21	1922	1942	GUGAAGUGUUUGGGGGAACUC	::..:::. :::.::::	CAACUCUUCCAGACAUUUCAG	Cleavage	reverse complement	1
miR395b-3p	*ZmFAR1-4*	5	-1	1	21	1922	1942	GUGAAGUGUUUGGGGGAACUC	::..:::. :::.::::	CAACUCUUCCAGACAUUUCAG	Cleavage	reverse complement	1
miR395c-3p	*ZmFAR1-14*	5	-1	1	21	1959	1979	GUGAAGUGUUUGGAGGAACUC	:::::.:::..::.: :::.	GAGUUUCUCUGAAUACUUUUA	Cleavage		1
miR395c-5p	*ZmFAR1-13*	5	-1	1	22	2842	2863	GUUCCCUGCAAACACUUCACCA	: ::::::: :: : :::	GUCUAAAGUGUUUGCUGAGAAG	Cleavage		1
miR395d-3p	*ZmFAR1-4*	5	-1	1	21	1922	1942	GUGAAGUGUUUGGGGGAACUC	::..:::. :::.::::	CAACUCUUCCAGACAUUUCAG	Cleavage	reverse complement	1
miR395d-5p	*ZmFAR1-2*	5	-1	1	22	3818	3839	GUUCUAUGCAAGCACUUCACGA	::::::: : .:: :::.	CCAAGAAGUGCUUCUAUCGAAU	Cleavage	reverse complement	1

miR395e-3p	*ZmFAR1-4*	5	-1	1	21	1922	1942	GUGAAGUGUUUGGGGGAACUC	::..:::. :::.::::	CAACUCUUCCAGACAUUUCAG	Cleavage	reverse complement	1
miR395f-3p	*ZmFAR1-4*	5	-1	1	21	1922	1942	GUGAAGUGUUUGGGGGAACUC	::..:::. :::.::::	CAACUCUUCCAGACAUUUCAG	Cleavage	reverse complement	1
miR395g-3p	*ZmFAR1-4*	5	-1	1	21	1922	1942	GUGAAGUGUUUGGGGGAACUC	::..:::. :::.::::	CAACUCUUCCAGACAUUUCAG	Cleavage	reverse complement	1
miR395g-5p	*ZmFAR1-2*	5	-1	1	22	3818	3839	GUUCUAUGCAAGCACUUCACGA	::::::: : .:: :::.	CCAAGAAGUGCUUCUAUCGAAU	Cleavage	reverse complement	1
miR395h-3p	*ZmFAR1-4*	5	-1	1	21	1922	1942	GUGAAGUGUUUGGGGGAACUC	::..:::. :::.::::	CAACUCUUCCAGACAUUUCAG	Cleavage	reverse complement	1
miR395i-3p	*ZmFAR1-4*	5	-1	1	21	1922	1942	GUGAAGUGUUUGGGGGAACUC	::..:::. :::.::::	CAACUCUUCCAGACAUUUCAG	Cleavage	reverse complement	1
miR395j-3p	*ZmFAR1-4*	5	-1	1	21	1922	1942	GUGAAGUGUUUGGGGGAACUC	::..:::. :::.::::	CAACUCUUCCAGACAUUUCAG	Cleavage	reverse complement	1
miR395k-5p	*ZmFAR1-15*	5	-1	1	22	587	608	GUUUCCUUCAAGCACUUCACAU	: :..::::: : :.::::..	CGGAGGGGUGCUGGAGGGAAGU	Translation	reverse complement	1
miR395l-3p	*ZmFAR1-14*	5	-1	1	21	1959	1979	GUGAAGUGUUUGGAGGAACUC	:::::.:::..::.: :::.	GAGUUUCUCUGAAUACUUUUA	Cleavage		1
miR395m-3p	*ZmFAR1-14*	5	-1	1	21	1959	1979	GUGAAGUGUUUGGAGGAACUC	:::::.:::..::.: :::.	GAGUUUCUCUGAAUACUUUUA	Cleavage		1
miR395n-3p	*ZmFAR1-4*	5	-1	1	21	1922	1942	GUGAAGUGUUUGGGGGAACUC	::..:::. :::.::::	CAACUCUUCCAGACAUUUCAG	Cleavage	reverse complement	1
miR395p-3p	*ZmFAR1-4*	5	-1	1	21	1922	1942	GUGAAGUGUUUGGGGGAACUC	::..:::. :::.::::	CAACUCUUCCAGACAUUUCAG	Cleavage	reverse complement	1
miR396f-3p	*ZmFAR1-6*	5	-1	1	21	814	834	GGUCAAGAAAGCUGUGGGAAG	:::: .:: : ::::::..	ACGCCCAGGGCCUUCUUGAUU	Translation		1
miR397a-5p	*ZmFAR1-11*	5	-1	1	21	2098	2118	UCAUUGAGCGCAGCGUUGAUG	::: .:: .:::..::::	GUACAAGAUUGUGCUUGAUGA	Cleavage		1
miR397b-5p	*ZmFAR1-11*	5	-1	1	21	2098	2118	UCAUUGAGCGCAGCGUUGAUG	::: .:: .:::..::::	GUACAAGAUUGUGCUUGAUGA	Cleavage		1
miR398a-3p	*ZmFAR1-1*	5	-1	1	21	2617	2637	UGUGUUCUCAGGUCGCCCCCG	:: :: :..::: :..::..	GAGGAGCCAUUUGAGGCAUG	Cleavage	reverse complement	1

miR398b-3p	*ZmFAR1-1*	5	-1	1	21	2617	2637	UGUGUUCU<br>CAGGUCGC<br>CCCCG	:: :: :..:::<br>:..:..	GAGGAGC<br>CAUUUGA<br>GGGCAUG	Cleavage	reverse complement	1
miR398b-5p	*ZmFAR1-4*	5	-1	1	21	6091	6111	GGGGCGGA<br>CUGGGAAC<br>ACAUG	: : :::.::::<br>.: :::.	UCUCUUU<br>UCUCAGU<br>UCUCCCU	Cleavage	reverse complement	1
miR399a-5p	*ZmFAR1-13*	5	-1	1	21	325	345	GUGCGGUU<br>CUCCUCUG<br>GCACG	: : :.::::.::.<br>:.:::	AGGGGCG<br>GAGGGGA<br>GCUGCAG	Cleavage		1
miR399d-5p	*ZmFAR1-10*	5	-1	1	21	8952	8972	GUGUGGCU<br>CUCCUCUG<br>GCAUG	::: ::: ::::::<br>:. .:.	CAUACCA<br>AAGGAGA<br>GCUCUAU	Cleavage		1
miR399d-5p	*ZmFAR1-13*	5	-1	1	21	325	345	GUGUGGCU<br>CUCCUCUG<br>GCAUG	: :.::::.:<br>:::...::	AGGGGCG<br>GAGGGGA<br>GCUGCAG	Cleavage		1
miR399f-3p	*ZmFAR1-10*	5	-1	1	21	281	301	UGCCAAAG<br>GAAAUUUG<br>CCCCG	::: ::::::<br>: ::: :	GCGGGGA<br>AAUUUCC<br>AUUGCCU	Cleavage		1
miR399g-3p	*ZmFAR1-8*	5	-1	1	21	849	869	UGCCAAAG<br>GGGAUUUG<br>CCCGG	: : :::...:<br>.::::::.	GAUGACU<br>AAUUUCU<br>UUUGGCG	Cleavage	reverse complement	1
miR399g-5p	*ZmFAR1-15*	5	-1	1	21	2219	2239	GGGCAACC<br>CCCCGUUG<br>GCAGG	: ::: :: :<br>::::::::	AGAGACA<br>AGGGUGG<br>GUUGCCC	Translatio<br>n	reverse complement	1
miR399h-5p	*ZmFAR1-10*	5	-1	1	21	8952	8972	GUGCAGUU<br>CUCCUCUG<br>GCACG	: : ::: ::::::.<br>:: .:.	CAUACCA<br>AAGGAGA<br>GCUCUAU	Cleavage		1
miR399i-5p	*ZmFAR1-10*	5	-1	1	21	8952	8972	GUGCGGCU<br>CUCCUCUG<br>GCAUG	::: ::: ::::::<br>:. .:.	CAUACCA<br>AAGGAGA<br>GCUCUAU	Cleavage		1
miR399j-5p	*ZmFAR1-16*	5	-1	1	21	881	901	AGGCAGCU<br>CUCCUCUG<br>GCAGG	: :.: :::::.<br>:: :.::	UGUUCUA<br>AAGGAGG<br>GCAGUCU	Cleavage		1
miR408a	*ZmFAR1-10*	5	-1	1	21	3688	3708	CUGCACUG<br>CCUCUUCC<br>CUGGC	:: :::<br>::::::::.:	GGAAGAC<br>AAGUGGC<br>AGUGCGG	Translatio<br>n		1
miR408b-3p	*ZmFAR1-10*	5	-1	1	21	3688	3708	CUGCACUG<br>CCUCUUCC<br>CUGGC	:: :::<br>::::::::.:	GGAAGAC<br>AAGUGGC<br>AGUGCGG	Translatio<br>n		1
miR482-3p	*ZmFAR1-10*	5	-1	1	20	3622	3641	UCUUCCUU<br>GUUCCUCC<br>CAUU	: : ::::::.:::<br>:.::.	ACUAGGA<br>GGAGCAA<br>AGGAGG	Cleavage		2
miR482-3p	*ZmFAR1-8*	5	-1	1	20	172	191	UCUUCCUU<br>GUUCCUCC<br>CAUU	.::: . ::::.:<br>:::.::	GAUGCAG<br>AGAACGA<br>GGAGGA	Cleavage	reverse complement	1
miR482-5p	*ZmFAR1-6*	5	-1	1	19	2536	2554	UGGGAGAU<br>GAAGGAGC<br>CUU	.:::: ..::...:.::<br>::	GAGGCAU<br>UUUUGUU<br>UCCCU	Cleavage		1

miR482-5p	*ZmFAR1-7*	5	-1	1	19	1087	1105	UGGGAGAU GAAGGAGC CUU	.::: :::::::. :..:	GAGGACC CUUCAUU UUUCU	Cleavage	reverse complement	1
miR529-5p	*ZmFAR1-3*	5	-1	1	21	3647	3667	AGAAGAGA GAGAGUAC AGCCU	::: :.:.: ::.::::.	GAGCUUC AUUUUCU UUCUUUC	Cleavage		1

Supplementary Table S4. The primer sequences used in qRT-PCR.

Primer name	Primer sequence
qACTIN-F	ATTGTCGGCAACTGGGATG
qACTIN-R	TCAGAGGAGCCTCGGTCAGC
qZmFAR1-1-F	CCAAGCAACTCCCACTAA
qZmFAR1-1-R	TACCCAGAACGGAACCAG
qZmFAR1-2-F	GATACCAACAAGCGAAAG
qZmFAR1-2-R	TGGTCTGGGATGAACTGG
qZmFAR1-3-F	GACAACCATACACAACCT
qZmFAR1-3-R	TCCGGACATGAGTGCCAA
qZmFAR1-4-F	GACGACTGGGTGGTCTAC
qZmFAR1-4-R	ATGCGTGCGCAGATGAG
qZmFAR1-5-F	ATCTACCTTGCCATCCACA
qZmFAR1-5-R	TTGTCAACCACGTCTCAAA
qZmFAR1-6-F	ATTTGAAGGAGCCTGACG
qZmFAR1-6-R	TGAGGAGGTAGGCGTAGT
qZmFAR1-7-F	GCTGCACAGCGTCCATAT
qZmFAR1-7-R	GGCAATGCGAACAGAGAA

qZmFAR1-8-F	AGCAGTCCTACTTCACCG
qZmFAR1-8-R	CCAGTCGGATAACCTTCC
qZmFAR1-9-F	AAGGTCAATCAGGTGTGG
qZmFAR1-9-R	AGAATAGACACATCGGCG
qZmFAR1-10-F	CTTCATCGACCACTAACT
qZmFAR1-10-R	ACCTTGCCATTGCAGGG
qZmFAR1-11-F	CGAACATGTAGGCTTCAG
qZmFAR1-11-R	CTAGGTGTGCCTGCTGAC
qZmFAR1-12-F	CTACTGGTATTAGGTTCG
qZmFAR1-12-R	GATTGGACTAGCACCTAC
qZmFAR1-13-F	CTGGACGGTGATGCGATG
qZmFAR1-13-R	CCTTAGGCATCGCACGTG
qZmFAR1-14-F	AAGAAGCCAGTTGAGCCTAGCA
qZmFAR1-14-R	CCCGGTCTGCACTAACAAT
qZmFAR1-15-F	CTACACGCATGAGGAGC
qZmFAR1-15-R	TCTGGGCGTCATCCTTG
qZmFAR1-16-F	TTAACAATGTCTACGGGTC
qZmFAR1-16-R	CCCATCCTTCATAGCACA
